# Ginseng and its active compounds in ovarian aging: mechanistic basis and translational prospects

**DOI:** 10.3389/fphar.2026.1755093

**Published:** 2026-01-26

**Authors:** Yan-Xin Li, Han-Zhi Zhong, Shao-Bin Wei

**Affiliations:** 1 School of Clinical Medicine, Chengdu University of Traditional Chinese Medicine, Chengdu, China; 2 Department of Gynecology, Hospital of Chengdu University of Traditional Chinese Medicine, Chengdu, China

**Keywords:** ovarian aging, *Panax ginseng*, ginsenosides, compound K, oxidative stress, inflammaging

## Abstract

Ovarian aging is characterized by follicular depletion and declining oocyte quality, encompassing both physiological age-related decline and pathological forms such as diminished ovarian reserve, premature ovarian insufficiency and premature ovarian failure. These changes are associated with long-term systemic comorbidities across the female life course, particularly in the context of estrogen deficiency. Ginseng as a botanical drug (*Panax ginseng* C.A.Mey.) and its active compounds, including ginsenosides Rg1, Rb1 and Rg3, the gut–derived metabolite Compound K and ginseng polysaccharides, have emerged as multitarget candidates for delaying ovarian aging-associated functional decline and supporting reproductive health. This review integrates preclinical evidence on how ginseng-related compounds attenuate oxidative stress, preserve mitochondrial function, support energy metabolism and modulate ovarian inflammaging and the senescence-associated secretory phenotype. They also rebalance apoptosis and autophagy, thereby supporting granulosa cell survival and follicle development. We summarize their regulatory effects on hypothalamic–pituitary–ovarian axis activity and on ovarian hormone receptor expression, which may help preserve ovarian endocrine function during aging. Across mechanistic domains, the most consistent ovary-relevant evidence converges on redox control and mitochondrial integrity and function, together with dampening of NF-κB and NLRP3-linked inflammatory signaling and SASP-associated features, whereas evidence for direct hypothalamic–pituitary modulation and for durable multisystem outcome modification remains more exploratory. Preclinical studies indicate that ginseng-related compounds can influence skeletal, cardiovascular, hepatic, metabolic and neurocognitive phenotypes that accompany estrogen deficiency. However, the evidence base remains heterogeneous and largely preclinical, and causal links to long-term functional reproductive outcomes are still limited. Interpretation of the existing literature is hampered by differences in botanical sources, processing methods, formulations, dosing regimens, treatment duration and routes of administration, which complicate evaluation of *in vivo* exposure and pharmacodynamic response, particularly for orally administered ginsenosides that undergo microbiota-mediated biotransformation and show inter-individual pharmacokinetic variability in some studies, with consequent uncertainty in dose relevance and exposure consistency across populations. Further progress toward clinical application may be facilitated by traceable and chemically defined ginseng preparations, exposure-guided oral dosing and rigorously designed clinical trials that better define efficacy, safety, plausible drug–drug interaction considerations and long-term reproductive and systemic outcomes with stage-stratified designs and prioritized functional outcome measures.

## Graphical Abstract

**Figure:**
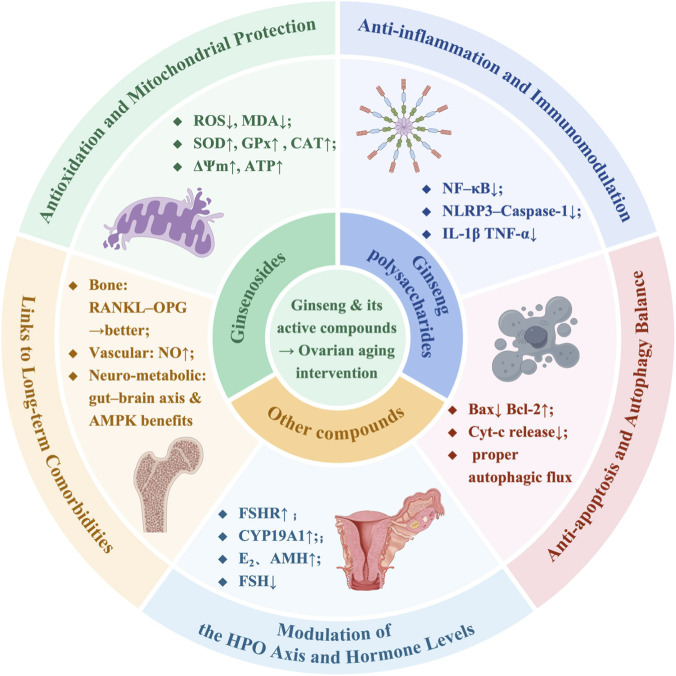

## 1 Introduction

Female reproductive aging reflects the finite, nonrenewable nature of the ovarian follicle pool and its age-related attrition, which together result in a progressive decline in both oocyte number and quality (Cavalcante et al., 2023). Follicular atresia is continuous and irreversible, and ovarian aging usually begins before the perimenopausal transition and often precedes aging in other organ systems. In mammals, the oocyte pool is established at birth and does not self-renew (Skinner, 2005). In humans, the ovarian follicle reserve is likewise fixed at birth and is gradually depleted through ongoing atresia, cyclic recruitment and ovulation (Richardson et al., 2014). As a result, reproductive capacity declines after 30 years of age, accelerates after 35 and falls more steeply after 40 (Broekmans et al., 2009). Ovarian aging includes physiological age-related decline and pathological conditions such as diminished ovarian reserve (DOR), premature ovarian insufficiency (POI), and premature ovarian failure (POF). Genetic and chromosomal abnormalities, immune dysregulation, iatrogenic injury, infection and environmental exposures can directly or indirectly impair oocyte quantity and competence (Li et al., 2021). The mechanisms that drive pathological decline remain complex and multifactorial, and thus pose a major challenge to female fertility and long-term health. Strategies that delay ovarian aging are therefore important not only for extending the reproductive window but also for reducing the burden of aging-related systemic diseases in aging populations.

Current clinical approaches include hormone replacement therapy (HRT), assisted reproductive technology and emerging stem cell-based strategies, although their benefits remain limited. HRT can alleviate menopausal and perimenopausal symptoms but has been associated with higher risks of breast cancer, stroke and venous thromboembolism (Collaborative Group on Hormonal Factors in Breast Cancer, 2019; Gu et al., 2014; Canonico et al., 2007). Assisted reproductive technology (ART) cannot fully overcome the age-related decline in fertility (Leridon, 2004). Women with ovarian aging frequently yield only a small number of oocytes or ultimately require donor oocytes (Chen et al., 2024). Stem cell therapies are still largely confined to preclinical studies and have not entered routine practice. Against this background, research on traditional Chinese medicine (TCM) has expanded globally, and botanical drugs of natural origin, multitarget pharmacology and relatively infrequent reports of adverse events are attracting increasing scientific attention.

Ginseng refers to the dried root and rhizome of *Panax ginseng* [Araliaceae; *P. ginseng* C.A.Mey.]. As a traditional qi-tonifying botanical drug, it has long been valued for broad pharmacological activities. Modern pharmacology has identified saponins, especially ginsenosides, as well as polysaccharides, amino acids, volatile oils, polyacetylenes and other compounds as key active compounds (Su et al., 2023). Accumulating evidence indicates that ginseng and its active compounds exert antioxidant, anti-inflammatory, and anti-apoptotic effects, modulate hormone secretion and improve ovarian functional indices in experimental models (He et al., 2017; Shang et al., 2023). Additional studies report benefits in cardiovascular, skeletal, metabolic, and neurocognitive domains, which suggests broader relevance for women’s health across the life course (Jafari et al., 2024; Jung et al., 2021; Park et al., 2022; Reay et al., 2010).

This review synthesizes recent advances on ginseng and its plant-derived metabolites, including gut-derived metabolites, in the context of ovarian aging. To clarify its novelty and positioning, we focus specifically on ovarian aging and ovarian functional decline, rather than ovarian function in general, and we adopt a female life-course perspective that links ovarian changes to multisystem outcomes associated with estrogen deficiency. We update the evidence base through 15 October 2025 by integrating studies across English and Chinese databases.

We organize the evidence across several mechanistic domains, including oxidative stress and mitochondrial homeostasis, regulation of ovarian inflammaging and the senescence-associated secretory phenotype (SASP), maintenance of granulosa cell survival and autophagy balance, modulation of the hypothalamic–pituitary–ovarian (HPO) axis, and potential links with long-term comorbidities. While the major mechanistic domains are broadly consistent with prior literature, our incremental contribution is a mechanism-guided synthesis that maps ginseng-related evidence onto an ovarian aging framework, integrates exposure-related considerations such as pharmacokinetics and microbiota-mediated biotransformation to contextualize variability in exposure, and summarizes safety signals and plausible drug–drug interaction (DDI) considerations relevant to long-term use. We also outline the material basis and translational features of ginseng, with emphasis on ginsenosides, the gut-derived metabolite Compound K (CK), and ginseng polysaccharides (GPS). Together, this review aims to provide an evaluative framework and research roadmap to guide mechanistic studies and translation directed at preserving ovarian function and delaying ovarian aging.

Notably, in clinical practice, POI is defined within a clear diagnostic framework based on persistent menstrual disturbance together with hormonal evidence of ovarian insufficiency (Panay et al., 2025). In contrast, DOR primarily denotes a functional phenotype of reduced ovarian reserve, commonly assessed using anti-Müllerian hormone (AMH), antral follicle count (AFC), and related tests, which are most useful for predicting oocyte number following stimulation rather than independently predicting reproductive potential (Medicine and American Society for Reproductive Medicine, 2020). In animal studies, POI and DOR are more appropriately treated as “POI-like” or “DOR-like” dysfunction states rather than being directly mapped to clinical diagnostic cut-offs. This is because estrous cyclicity differs from human menstrual cyclicity in physiology and assessment, and hormone baselines and dynamics vary by species and assay, making universally applicable cut-offs difficult to standardize across studies (Lu et al., 2022; Dai et al., 2023; Balough et al., 2024). Moreover, many commonly used models are induced by chemotherapy, toxicants, or immune perturbations, capturing specific etiologic or injury processes that may not be equivalent to the heterogeneous clinical entities defined in humans (Pouladvand et al., 2024; Dai et al., 2023; Lu et al., 2022). *In vitro* experiments cannot define POI or DOR in a diagnostic sense because these entities are fundamentally system-level phenotypes involving follicle pool dynamics, ovulation, and endocrine feedback regulation within the HPO axis (Valera et al., 2025). Such features cannot be fully recapitulated by isolated cells or simplified culture systems. Accordingly, *in vitro* evidence is best interpreted as mechanistic support relevant to ovarian aging or ovarian dysfunction contexts, rather than as a basis for assigning POI or DOR diagnostic categories (Park et al., 2024). Although clinical diagnostic categories should not be transplanted directly onto *in vivo* and *in vitro* systems, model establishment and phenotyping for ovarian aging and ovarian dysfunction have relatively consistent, reusable endpoint sets. For physiologic reproductive aging models, commonly used criteria include prolonged or irregular estrous cyclicity, age-associated shifts in follicle number and stage distribution, ovarian histologic remodeling, and age-dependent changes in reproductive hormones and reserve-related markers, ideally complemented by reproductive performance outcomes (Balough et al., 2024; Lu et al., 2022). For chemically or drug-induced ovarian dysfunction models, evaluation commonly includes disrupted estrous cyclicity, reduced E2 and AMH, elevated FSH, increased follicular atresia, and depletion of the follicle pool (Stringer et al., 2018; Jiao and Zhou, 2025). Methodological recommendations for follicle counting and histopathologic assessment further support cross-study comparability (Regan et al., 2005).

## 2 Literature search and study selection

We searched PubMed, Embase, and CNKI from database inception to 15 October 2025, and additionally consulted *the Pharmacopoeia of the People’s Republic of China* for official botanical and quality-control information on *Panax ginseng* C.A. Mey. Searches were conducted using controlled vocabulary (MeSH in PubMed and Emtree in Embase) and free-text keywords, and combined two concept blocks: (1) Ovarian aging or ovarian functional decline (including DOR, POI, and POF); (2) Ginseng-related interventions (ginseng, *Panax ginseng*, ginsenosides, Rg1, Rb1, Rg3, Compound K, and ginseng polysaccharides). The complete database-specific search strings and field tags are provided in Supplementary Table S1. We limited results to articles published in English or Chinese. In addition, we hand-searched reference lists of relevant reviews and included studies to identify additional eligible records.

All records retrieved from the database searches were exported to a reference manager for deduplication. Titles and abstracts were screened for relevance, and potentially eligible articles underwent full-text assessment. Study inclusion was determined based on the predefined eligibility criteria described below. For included studies, we summarized key information on study design (*in vivo* or *in vitro*), model characteristics, ginseng preparation or metabolite identity and purity, dosing regimen and exposure duration, and outcomes across oxidative stress, mitochondrial function, inflammatory or senescence-associated phenotypes, apoptosis or autophagy, HPO axis measures, and where available, reproductive outcomes or estrogen deficiency related systemic phenotypes.

To aid readability, Table 1 is arranged to allow rapid comparison across the included studies. It consolidates study type and model features together with intervention characterization, dose or concentration, route, duration, and the main measures reported, so that similarities and differences can be identified at a glance. Across the included literature, the evidence base is predominantly preclinical and spans ovarian aging-related ovarian models, granulosa cell and follicle culture systems, and selected immune cell systems used to probe inflammatory pathways. Table 1 serves as a navigational summary that supports interpretation of the mechanistic synthesis presented later in this review, where the included findings are organized across key mechanistic domains. To provide a basic assessment of study quality, we conducted a simplified reporting-based checklist of key items sensitive to bias for each included study. This checklist focused on whether control conditions were clearly described, including negative controls and matched vehicle or solvent controls when applicable, whether exposure details were sufficiently reported, including dose or final concentration, route, frequency, timing, and duration, and whether the ginseng intervention was adequately characterized, including extract preparation and chemical profiling, or compound source and purity. Each item was coded as R, P, or NR to reflect reporting completeness and its likely impact on interpretability, and this coding was used as a reporting checklist rather than a subjective risk-of-bias rating. The checklist results are summarized in Supplementary Table S2, which provides a study-by-study snapshot of reporting completeness across the key items.

**TABLE 1:** Ginseng and its active compounds used in ovarian aging–related models.

Mechanistic category	Metabolites/Extracts	Preparation method	Identification method	Controls	Cells/Animals	Model	Dose (concentration)	Duration of administration	Mechanisms	References
Antioxidation and mitochondrial protection	Ginsenoside Rg1 (Rg1)	Rg1 (Cat. RSZD-121106) was purchased from Xi’an Haoxuan Biological Technology Co., Ltd.; the compound was dissolved in ddH_2_O to 20 mg/mL and sterilized by filtration.	Purity 98.3% (as provided by the supplier)	Saline group; D-gal group; D-gal + Rg1 group; Rg1 group	C57BL/6 female mice (3-month-old)	D-gal–induced POF(D-gal 200 mg/kg/d, s.c., 42 days)	Rg1 20 mg/kg/day,intraperitoneal (i.p.); started on day 15 after first D-gal injection。	Rg1 for 28 days; D-gal for 42 days	T-SOD ↑; GSH-Px ↑; MDA ↓; IL-1β ↓; TNF-α ↓; IL-6 ↓; with attenuated oxidative and inflammatory damage in ovarian tissue.	He et al. (2017).
	Ginsenoside Rg1 (Rg1)	Rg1 was purchased from Yuanye Biological Technologies (Shanghai, China)	Purity ≥98% (supplier-reported)	Vehicle control (0.1% DMSO); Rg1 alone (20 μg/mL, 24 h); H_2_O_2_ alone (200 μM, 24 h); Rg1 pretreatment 24 h, then H_2_O_2_ 24 h (20 μg/mL; 200 μM)	KGN human ovarian granulosa cells	H_2_O_2_-induced oxidative stress in KGN cells (200 μM, 24 h)	Rg1 20 μg/mL (*in vitro*)	24 h (Rg1 pretreatment) + 24 h (H_2_O_2_ exposure) in the combination group; 24 h in single-agent groups	ROS ↓; mitochondrial ROS ↓; MDA ↓; GSH ↑; ATP ↑; ΔΨm restored; with improved mitochondrial morphology and alleviated oxidative stress via modulation of the PI3K–Akt–mTOR pathway.	Li et al. (2025).
	Ginsenoside Rb1 (Rb1)	Rb1 stock solution was prepared in DMSO; working solutions were made freshly with serum-free medium before each experiment.	Purity >98% (supplier-reported; catalog PCS0759)	Control (no H_2_O_2_, no Rb1); H_2_O_2_ alone; Rb1 alone; Rb1 pretreatment followed by H_2_O_2_ exposure	KGN human ovarian granulosa cell line; human granulosa-lutein (hGL) cells from young (≤30 years) and aged (≥38 years) women.	H_2_O_2_-induced oxidative stress in KGN cells (viability assay used 2 h exposure)	Rb1 10 μM (*in vitro*; identified as the most effective concentration)	Rb1 pretreatment (hours, assay-dependent) followed by H_2_O_2_ exposure (e.g., 2 h for viability)	ROS ↓; LDH ↓; MDA ↓; SOD ↑; mitochondrial membrane potential (ΔΨm) ↑; cleaved caspase-3 ↓; cleaved caspase-9 ↓; p-Akt (Ser473) ↑; p-FoxO1 (Ser256) ↑; p-Akt–FoxO1 interaction ↑; FoxO1 nuclear translocation ↓.	Zhou et al. (2022).
	Ginsenoside Rb1 (Rb1)	Rb1 (purity >98%; catalog PCS0759) was freshly prepared in saline for injection.	Purity >98% (supplier-reported; catalog PCS0759)	Vehicle-treated young mice; Rb1-treated young mice; vehicle-treated old mice; Rb1-treated old mice.	ICR mice (young and old)	Aging-related ovarian changes in ICR mice	Rb1 10 mg/kg/day, intraperitoneal	Daily administration for 2 weeks	ROS ↓; MDA ↓; SOD ↑; mitochondrial membrane potential (ΔΨm) ↑; cleaved caspase-3 ↓; cleaved caspase-9 ↓; p-Akt (Ser473) ↑; p-FoxO1 (Ser256) ↑.	Zhou et al. (2022).
	Panax ginseng (PG) powder	PG powder (gifted by Barij Essence Pharmaceutical Co.) dissolved in 1% carboxymethyl cellulose (CMC)–saline; administered by gastric gavage	NR	Control (no drug); Sham (1% CMC–saline, oral); Nicotine only (0.6 mg/kg, i.p.); Nicotine + PG 0.5 g/kg (oral); Nicotine + PG 1 g/kg (oral)	Adult female NMRI mice, 8–10 weeks, 35 ± 5 g	Nicotine-induced ovarian impairment (nicotine 0.6 mg/kg/day, intraperitoneal)	PG 0.5 g/kg/day or 1 g/kg/day, oral gavage	30 days (once daily)	Estradiol ↑; Progesterone ↑; FSH ↑; LH ↑; Serum MDA ↓; Tissue MDA ↓; Serum CAT ↑; Tissue SOD ↑ (serum SOD ↑ trend); Ki-67 proliferative index (granulosa/theca/stroma) ↑; Bax ↓; Cyt c ↓; Bcl-2 ↑; Bax/Bcl-2 ratio ↓; Healthy follicles ↑; Atretic follicles ↓; Corpus luteum ↑.	Faghani et al. (2022).
Anti-inflammation and immunomodulation	Rare ginsenosides (heat-transformed saponins, HTS)	HTS prepared in-house by thermal conversion (per Xue et al., 2020) from P. quinquefolius stem/leaf saponins (Jilin Hongzhou Biotechnology); reagents otherwise per methods	HPLC profiling (Shimadzu; YMC ODS-Pack 4.6 × 250 mm; water/acetonitrile gradient) identifying Rg6, F4, Rh4, Rg3(S), Rg3(R), Rk1, Rg5, Rh2; total saponin content by vanillin–glacial acetic acid–perchloric acid method; HTS content 85.57%	Control (untreated KGN); CP only (320 μM); CP + HTS low (5 mg/L); CP + HTS medium (10 mg/L); CP + HTS high (20 mg/L)	KGN human granulosa-like cells	CP-induced injury/oxidative stress (CP 320 μM)	HTS 5, 10, or 20 mg/L (i.e., μg/mL) *in vitro*	CP 24 h, then HTS 24 h (per assay workflow)	Cell survival ↑; apoptosis ↓ (Annexin V/PI); ROS ↓; SOD ↑ or normalized (supernatant); GSH ↑; Ki67+ cells ↑; p-p38 MAPK ↓; p-NF-κB p65 ↓; TNF-α ↓; IL-1β ↓; IL-6 mRNA ↓; AMH ↑; E_2_ ↑; FSH ↓.	Tao et al. (2024).
	Rare ginsenosides (heat-transformed saponins, HTS) from Panax quinquefolius stems/leaves	HTS prepared in-house by thermal conversion (per Xue et al., 2020) from P. quinquefolius stem/leaf saponins (Jilin Hongzhou Biotechnology); reagents otherwise per methods	HPLC profiling (Shimadzu; YMC ODS-Pack 4.6 × 250 mm; water/acetonitrile gradient) identifying Rg6, F4, Rh4, Rg3(S), Rg3(R), Rk1, Rg5, Rh2; total saponin content by vanillin–glacial acetic acid–perchloric acid method; HTS content 85.57%	Normal control (saline gavage); CP model (CP only); HTS low-dose (L-HTS); HTS medium-dose (M-HTS); HTS high-dose (H-HTS)	Female Sprague–Dawley rats (6-week-old)	Cyclophosphamide-induced POF (CP 50 mg/kg/day, intraperitoneal, for 5 consecutive days)	HTS 150, 300, or 600 mg/kg/day, oral gavage	CP 5 consecutive days; HTS daily by oral gavage	Serum/tissue MDA ↓; GSH ↑; serum IL-6 ↓; serum TNF-α ↓; ovarian NF-κB p65 (IHC) ↓; p-p38 MAPK ↓; p-NF-κB p65 ↓; IL-1β ↓; Ki67 (ovarian GCs) ↑; estrous cyclicity improved; AMH ↑; E_2_ ↑; FSH ↓; growing follicles ↑; atretic follicles ↓; ovarian index ↑.	Tao et al. (2024).
	Ginsenoside Rg3 (Rg3)	Rg3 (purity ≥98%, SML0184) reconstituted in DMSO at 5 mg/mL; diluted in culture medium to indicated concentrations	Purity ≥98% (supplier-reported)	Vehicle control; LPS priming only; NLRP3 activation controls (LPS for 4 h, then ATP for 1 h; or LPS for 4 h, then nigericin for 1 h; or LPS for 4 h, then alum for 4 h); AIM2 activation control (LPS for 4 h, then poly (dA:dT) for 8 h); NLRC4 activation control (LPS for 4 h, then flagellin for 8 h); non-canonical activation (Pam3CSK4 for 4 h, then cytosolic LPS for 6 h); NLRP3-deficient THP-1 controls	THP-1 (human); THP-1 NLRP3-deficient; J774.A1 (mouse); RAW264.7 and RAW264.7-ASC; mouse BMDMs; HEK293T (reconstitution system)	LPS-primed inflammasome activation models (NLRP3, AIM2, NLRC4; canonical and non-canonical)	Rg3 added after LPS priming for 1 h at indicated doses (e.g., 10 μg/mL in immunoblot assays)	LPS priming 4 h; Rg3 for 1 h; subsequent stimuli typically 1 h (ATP, nigericin) or 4 h (alum); transfection-based stimuli 6–8 h	IL-1β ↓; IL-18 ↓; caspase-1 p20 ↓; LDH release ↓; TNF-α ns; IL-6 ns; NEK7–NLRP3 interaction ↓; NLRP3–ASC interaction ↓; ASC oligomerization ↓; ASC speck formation ↓; NLRP3 oligomerization ↓; NLRP3 ATPase activity ns; mitochondrial ROS ns; mitochondrial membrane potential (ΔΨm) ns; pro-IL-1β (priming) ↓.	Shi et al. (2020).
	Ginsenoside Rg3 (Rg3)	Rg3 (purity ≥98%, SML0184) reconstituted in DMSO at 5 mg/mL; diluted in culture medium to indicated concentrations	Purity ≥98% (supplier-reported)	PBS/vehicle plus LPS; Rg3 pretreatment plus LPS; survival cohort with or without Rg3 after high-dose LPS	C57BL/6J mice (8–10 weeks)	LPS-induced peritonitis (10 mg/kg, intraperitoneal) and LPS-induced endotoxic shock (20 mg/kg, intraperitoneal)	Rg3 10 mg/kg, intraperitoneal	Peritonitis protocol: Rg3 once daily for 3 days before LPS; sampling 6 h after LPS. Endotoxic shock protocol: Rg3 every 24 h for 72 h; survival monitored up to 72 h	Serum IL-1β ↓; serum TNF-α ↓ (slight); serum IL-6 ns; peritoneal IL-1β ↓; peritoneal TNF-α ↓; peritoneal IL-6 ns; splenomegaly ↓; splenic inflammatory cell accumulation ↓; survival ↑.	Shi et al. (2020).
Anti-inflammation and immunomodulation	Compound K (CK), a metabolite of ginsenoside Rb1.	CK (purity 97%) obtained from Ambo Institute (Daejeon, Korea)	Purity 97% (supplier-reported)	Vehicle control; LPS alone (1 μg/mL, 24 h); CK alone (0–10 μM, 24 h); LPS + CK (e.g., 10 μM). Comparator inhibitors: LY294002 (25 μM) and BN82002 (25 μM).	RAW264.7 murine macrophage-like cells.	LPS-activated inflammatory response (1 μg/mL, 24 h)	CK 0–10 μM (key effects at 5–10 μM)	LPS 24 h with or without CK 24 h	iNOS ↓; TNF-α ↓; p-AKT1 ↓, no effect on AKT2; p-IKKα/β ↓; with suppression of AKT1–IKK–NF-κB inflammatory signaling in macrophages.	Lee et al. (2019).
	Compound K (CK), a metabolite of ginsenoside Rb1.	CK (purity 97%) obtained from Ambo Institute (Daejeon, Korea)	Purity 97% (supplier-reported)	Vector control; NF-κB luciferase assays with MyD88 or TAK1 overexpression ± CK; Src/AKT1/AKT2 overexpression ± CK	HEK293 cells	MyD88/TAK1-driven NF-κB activation; Src-driven AKT activation; AKT1 vs. AKT2 overexpression.	CK 0–10 μM (key effects at 5–10 μM)	NF-κB reporter: transfection 24 h, then CK 24 h; Src/AKT experiments: transfection 48 h, then CK 24 h	p-AKT1 ↓, no effect on AKT2; AOX1 ↓; iNOS ↓; NF-κB luciferase activity ↓; confirming selective inhibition of the AKT1–NF-κB axis.	Lee et al. (2019).
	Panax ginseng extract (PGE; “G115”) from Pharmaton Company (Switzerland)	PGE stock prepared in DMSO (10 g/mL) and diluted in culture medium to final concentrations of 50, 100, and 500 μg/mL.	Composition referenced from a prior HPLC analysis of Pharmaton G115 (not measured in this study): Rg1 4.61 ± 0.43 mg/g; Rb1 1.39 ± 0.12 mg/g; Rb2 11.59 ± 1.30 mg/g; Rf 2.36 ± 0.25 mg/g; Rd 9.06 ± 1.05 mg/g; Rc 3.99 ± 0.20 mg/g; Re 9.59 ± 0.85 mg/g (HPLC; ref. 18 in the article).	Control (no PGE); Experimental group 1 (50 μg/mL PGE); Experimental group 2 (100 μg/mL PGE); Experimental group 3 (500 μg/mL PGE)	Isolated preantral follicles from 14-day-old female NMRI mice; individually encapsulated in alginate	3D alginate encapsulation culture of mouse preantral follicles (α-MEM-based medium with supplements; *in vitro* growth and maturation)	PGE 0/50/100/500 μg/mL	12-day culture; hCG added on day 12 and outcomes assessed ∼24 h later (MII scoring, hormones, ROS)	ROS in MII oocytes ↓; with reduced oxidative stress burden during 3D preantral follicle culture and protection of oocyte quality.	Majdi Seghinsara et al. (2019).
Anti-apoptosis and autophagy balance	Ginsenoside Rg1 (Rg1)	Rg1 was purchased from Yuanye Biological Technologies (Shanghai, China)	Purity ≥98% (supplier-reported)	Vehicle control (0.1% DMSO); Rg1 alone (20 μg/mL, 24 h); H_2_O_2_ alone (200 μM, 24 h); Rg1 pretreatment 24 h, then H_2_O_2_ 24 h (20 μg/mL; 200 μM)	KGN human ovarian granulosa cells	H_2_O_2_-induced oxidative stress in KGN cells (200 μM, 24 h)	Rg1 20 μg/mL (*in vitro*)	24 h (Rg1 pretreatment) + 24 h (H_2_O_2_ exposure) in the combination group; 24 h in single-agent groups	Bcl-2 ↑; Bax ↓; Caspase-3 ↓; Beclin1 ↓; LC3B-II ↓; PINK1 ↓; Parkin ↓; with reduced apoptosis and excessive mitophagy and preserved autophagic homeostasis via the PI3K–Akt–mTOR pathway, improving granulosa cell survival.	Li et al. (2025).
	Ginsenoside Rg1 (Rg1)	Rg1 purchased from Meilunbio (purity >98%, batch MB6863); solvent for Rg1 not reported; administered intraperitoneally.	Purity >98% (supplier-reported)	PBS group; D-gal group; Rg1 group (D-gal plus Rg1 from day 15)	Female SPF BALB/c mice, 6–8 weeks (≈18.1 ± 0.8 g)	D-gal–induced POF mouse model.	D-gal 200 mg/kg/day, subcutaneous, for 42 days; Rg1 20 mg/kg/day, intraperitoneal, starting on day 15, for 28 days.	D-gal 42 days; Rg1 28 days (day 15–42)	Estrous cyclicity improved; ovarian SA-β-Gal ↓; p16INK4a (mRNA/protein) ↓; LC3-II (autophagy marker) ↑; PI3K/Akt/mTOR/S6k proteins ↓ vs. D-gal; Akt/mTOR/S6k mRNA ↓ vs. D-gal — indicating modulation of the PI3K–Akt–mTOR autophagy pathway.	Liu et al. (2020a).
Anti-apoptosis and autophagy balance	Ginsenoside Rg1 (Rg1)	Rg1 (Cat. RSZD-121106) was purchased from Xi’an Haoxuan Biological Technology Co., Ltd.; the compound was dissolved in ddH_2_O to 20 mg/mL and sterilized by filtration.	Purity 98.3% (as provided by the supplier)	saline group; D-gal group; D-gal + Rg1 group; Rg1 group	C57BL/6 female mice (3-month-old)	D-gal–induced POF (D-gal 200 mg/kg/d, s.c., 42 days)	Rg1 20 mg/kg/day,intraperitoneal (i.p.); started on day 15 after first D-gal injection。	Rg1 for 28 days; D-gal for 42 days	p53, p21, p19, p16 ↓; with reduced granulosa cell senescence and apoptosis and alleviated ovarian pathological damage.	He et al. (2017).
	Ginsenoside Rg3 (Rg3)	Rg3 (purity >98%) purchased from Chengdu Herbpurify; cells were pretreated with Rg3 for 24 h before H_2_O_2_ exposure; solvent/stock not reported.	Purity >98% (supplier-reported)	Control; H_2_O_2_ model; H_2_O_2_ + Rg3; Rg3 alone	Human ovarian granulosa cell line KGN (gift from Peking University Third Hospital)	H_2_O_2_-induced oxidative stress in KGN; 25–400 μM H_2_O_2_ tested for 2 h; 200 μM for 2 h selected for modeling	Rg3 screening at 2.5, 5, 10, 20, 40 mg/L with 24 h pretreatment; 10 mg/L Rg3 used for subsequent assays; H_2_O_2_ 200 μM for 2 h.	Rg3 24 h pretreatment; H_2_O_2_ 2 h exposure.	Cell viability ↑; ROS ↓; apoptosis rate ↓ (Annexin V/PI); cytochrome c ↓; Bcl-2/BAX ↑ These changes indicate attenuation of mitochondria-mediated apoptosis under oxidative stress.	Zhou et al. (2021).
	Ginsenoside Rb1 (Rb1)	Rb1 administered by oral gavage; solvent/stock and supplier NR.	Purity NR	Control group (no treatment); Model group (cyclophosphamide only); Treatment group (cyclophosphamide + Rb1)	Female Wistar rats, 2–3 months old, ∼200 ± 20 g, normal estrous cycles.	Cyclophosphamide-induced premature ovarian failure: loading 50 mg/kg i.p. once, then 8 mg/kg/day i.p. for 14 days.	Rb1 25 mg/kg/day, oral gavage (treatment group)	Cyclophosphamide: 15 days total (1-day loading +14 days maintenance). Rb1: 4 weeks (once daily)	Bcl-2 ↑; Bax ↓; Bcl-2/Bax ratio ↑; granulosa cell apoptosis ↓ (IHC and Western blot).	Tang et al. (2014).
	Compound K (CK), a metabolite of ginsenoside Rb1.	CK (purity 97%) obtained from Ambo Institute (Daejeon, Korea)	Purity 97% (supplier-reported)	Vehicle control; LPS alone (1 μg/mL, 24 h); CK alone (0–10 μM, 24 h); LPS + CK (e.g., 10 μM). Comparator inhibitors: LY294002 (25 μM) and BN82002 (25 μM).	RAW264.7 murine macrophage-like cells.	LPS-activated inflammatory response (1 μg/mL, 24 h)	CK 0–10 μM (key effects at 5–10 μM)	LPS 24 h with or without CK 24 h	Cell viability preserved at 0–10 μM CK; LPS-induced morphological damage alleviated; with protection against macrophage inflammatory injury and maintenance of cell survival.	Lee et al. (2019).
	Ginseng polysaccharides (GPS)	GPS added to culture medium at 30 ng/mL; supplier/stock NR	NR	37 °C control; cold-stress groups at 0, −15, −25 °C; with or without GPS.	Rat oocytes isolated from ovaries after PMSG priming; two preparations: follicle-enclosed oocytes and cumulus-enclosed oocytes.	Cold-stress exposure at 0, −15, −25 °C for 10 min, then rewarming at 37 °C for further culture.	GPS 30 ng/mL *in vitro*.	GPS pre-incubation 8–12 h (follicle-enclosed) or 8–10 h (cumulus-enclosed); cold stress 10 min; post-rewarming culture 6–8 h (follicle-enclosed) or 4–8 h (cumulus-enclosed)	For follicle-enclosed oocytes: GVBD% ↑ under cold stress with GPS; for cumulus-enclosed oocytes: GVBD% ns with GPS.	Feng et al. (2007).
	Ginseng polysaccharides (GPS)	GPS added to culture medium at 30 ng/mL; supplier/stock NR	NR	37 °C control; cold-stress groups at 0, −15, −25 °C; with or without GPS.	Rat ovarian granulosa cells isolated from ovaries	Cold-stress exposure at 0, −15, −25 °C for 10 min, then rewarming at 37 °C for further culture.	GPS 30 ng/mL *in vitro*.	For DNA/protein synthesis assays: GPS with ^3^H-TdR or ^3^H-Leu for 48 h; in cold-stress workflow: culture 24 h, cold stress, rewarming 37 °C, then culture 24 h before collection.	^3^H-TdR incorporation ↑; ^3^H-Leu incorporation ↑; indicates enhanced DNA replication and protein synthesis under cold stress with GPS.	Feng et al. (2007).
Anti-apoptosis and autophagy balance	Ginseng polysaccharides (GPS)	GPS (70%) purchased from Shanghai Yuanye Biotech (batch C12N9Y74733); dissolved in DMEM/F12 with fetal bovine serum; cells treated for 24 h.	Purity 70% (supplier-reported)	Control (0 μg/mL GPS); GPS 20, 40, 60, 80 μg/mL for 24 h.	Primary bovine ovarian granulosa cells isolated from abattoir ovaries; cultured in DMEM/F12 at 37 °C with 5% CO_2_.	*In vitro* culture model assessing proliferation and E_2_ secretion under GPS treatment.	Screening range 0–80 μg/mL; 40 μg/mL selected for downstream assays.	GPS 24 h	Cell viability ↑; S-phase cell ratio ↑; PCNA, CDC42, CDK2, CDK4, Cyclin A2, Cyclin D2 mRNA ↑; PCNA, CDK2 protein ↑; with enhanced proliferation of bovine ovarian granulosa cells.	Chen et al. (2025).
Modulation of the HPO axis and hormone levels	Ginsenoside Rg1 (Rg1)	Rg1 purchased from Meilunbio (purity >98%, batch MB6863); administered intraperitoneally; solvent/stock not reported.	Purity >98% (supplier-reported)	PBS group; D-gal group; Rg1 group (D-gal plus Rg1 from day 15).	Female SPF BALB/c mice, 6–8 weeks, ∼21.6 ± 1.7 g	D-gal–induced POF model by subcutaneous injection	D-gal 200 mg/kg/day, subcutaneous, 42 days; Rg1 20 mg/kg/day, intraperitoneal, starting on day 15, 28 days.	D-gal 42 days; Rg1 28 days (day 15–42)	SIRT1 mRNA ↑; SIRT1 protein ↑; serum E_2_ ↑; serum LH ↑; serum FSH ↓; serum SOD ↑; serum CAT ↑; ovarian follicle numbers ↑; atretic follicles ↓; corpus luteum vacuolization ↓; body-weight gain rate ↑; ovarian weight index ↑.	Liu et al. (2020b).
	Ginsenoside Rg1 (Rg1)	Rg1 (Cat. RSZD-121106) was purchased from Xi’an Haoxuan Biological Technology Co., Ltd.; the compound was dissolved in ddH_2_O to 20 mg/mL and sterilized by filtration.	Purity 98.3% (as provided by the supplier)	saline group; D-gal group; D-gal + Rg1 group; Rg1 group	C57BL/6 female mice (3-month-old)	D-gal–induced POF(D-gal 200 mg/kg/d, s.c., 42 days)	Rg1 20 mg/kg/day,intraperitoneal (i.p.); started on day 15 after first D-gal injection.	Rg1 for 28 days; D-gal for 42 days	FSHR ↑; with improved estrous cyclicity and fertility and enhanced ovarian responsiveness to gonadotrophins.	He et al. (2017).
	Panax ginseng extract (PGE; “G115”) from Pharmaton Company (Switzerland)	PGE stock prepared in DMSO (10 g/mL) and diluted in culture medium to final concentrations of 50, 100, and 500 μg/mL.	Composition referenced from a prior HPLC analysis of Pharmaton G115 (not measured in this study): Rg1 4.61 ± 0.43 mg/g; Rb1 1.39 ± 0.12 mg/g; Rb2 11.59 ± 1.30 mg/g; Rf 2.36 ± 0.25 mg/g; Rd 9.06 ± 1.05 mg/g; Rc 3.99 ± 0.20 mg/g; Re 9.59 ± 0.85 mg/g (HPLC; ref. 18 in the article).	Control (no PGE); Experimental group 1 (50 μg/mL PGE); Experimental group 2 (100 μg/mL PGE); Experimental group 3 (500 μg/mL PGE)	Isolated preantral follicles from 14-day-old female NMRI mice; individually encapsulated in alginate	3D alginate encapsulation culture of mouse preantral follicles (α-MEM-based medium with supplements; *in* *vitro* growth and maturation)	PGE 0/50/100/500 μg/mL	12-day culture; hCG added on day 12 and outcomes assessed ∼24 h later (MII scoring, hormones, ROS)	PCNA mRNA ↑; FSHR mRNA ↑; E_2_ ↑; P4 ↑; DHEA ↑; with enhanced follicle survival, antrum formation, oocyte maturation and steroidogenesis in 3D-cultured preantral follicles.	Majdi Seghinsara et al. (2019).
	Ginseng polysaccharides (GPS)	GPS (70%) purchased from Shanghai Yuanye Biotech (batch C12N9Y74733); dissolved in DMEM/F12 with fetal bovine serum; cells treated for 24 h.	Purity 70% (supplier-reported)	Control (0 μg/mL GPS); GPS 20, 40, 60, 80 μg/mL for 24 h.	Primary bovine ovarian granulosa cells isolated from abattoir ovaries; cultured in DMEM/F12 at 37 °C with 5% CO_2_.	*In vitro* culture model assessing proliferation and E_2_ secretion under GPS treatment.	Screening range 0–80 μg/mL; 40 μg/mL selected for downstream assays.	GPS 24 h	E_2_ secretion ↑; CYP11A1, CYP19A1, 3β-HSD, STAR mRNA ↑; CYP11A1, STAR protein ↑; with activated steroidogenic pathway and improved estrogen biosynthesis in granulosa cells.	Chen et al. (2025).
	Ginseng polysaccharides (GPS)	GPS provided by Jilin University (stock 2 g/L); added to oocyte culture medium (dose details NR for concentration units)	NR	Oocyte cultures with or without GPS; viability assessed after incubation.	Oocytes isolated from adult female rat ovaries (PMSG-primed)	*In vitro* oocyte culture to assess survival under GPS treatment	Various GPS doses tested; threshold effect observed starting at ≥20 μL of GPS stock added; exact concentration NR.	GPS incubation 8–10 h before viability assessment.	Oocyte growth inhibitory rate ↓; viability ↑; dose–response relationship observed within the tested range.	Sun et al. (2007).
Modulation of the HPO axis and hormone levels	Panax ginseng decoction (PGD)	Dried P. ginseng was decocted twice in water (soak 1 h; boil/reflux 1.5 h each), filtrates merged and concentrated under reduced pressure to 0.3 g/mL PGD	Botanical authentication with voucher YHZY20151107; extract composition referenced from prior ESI-MS profiling of ginsenosides (this study did not newly quantify purity).	Sham-operated group (SG); estrogen-decline model group (EG; OVX); positive group estradiol valerate 0.15 mg/kg; PGD group 20 mL/kg intragastrically	Female Sprague–Dawley rats (220–250 g)	Ovariectomy-induced estrogen decline (dorsal OVX; success confirmed by ≥ 5 days leukocyte smears)	Dose (concentration): PGD 20 mL/kg, intragastric (i.g.); PGD concentration 0.3 g/mL.	8 weeks (daily administration stated across the 8-week treatment period; samples collected at week 8)	Estrous cyclicity improved; E_2_ ↑; LH ↓; uterine index ↑; urinary metabolomic shifts normalized toward sham, implicating steroid hormone metabolism, fatty acid biosynthesis, TCA cycle, and tryptophan metabolism.	Lin et al. (2018).

Data are summarized from *in vivo* and *in vitro* studies of ginseng and its active compounds in models of ovarian aging or related ovarian dysfunction. ↑ and ↓ indicate increases or decreases relative to the corresponding control or model groups; NR, not reported. D-gal, D-galactose; POI, premature ovarian insufficiency; POF, premature ovarian failure.

Predefined inclusion criteria: (1) Study populations or experimental models had to be relevant to ovarian aging or decline in ovarian function, including DOR, POI and POF, or to estrogen deficiency related states in which skeletal, cardiovascular, metabolic or neurocognitive comorbidities were evaluated; (2) Interventions involving ginseng or clearly defined active compounds and metabolites, such as Ginsenoside Rg1 (Rg1), Ginsenoside Rb1 (Rb1), Ginsenoside Rg3 (Rg3), CK, and GPS; (3) Articles had to be published in Chinese or English; (4) Outcomes needed to include at least one endpoint related to redox status, mitochondrial function, inflammatory or senescence-associated phenotypes, apoptosis or autophagy, regulation of the HPO axis or endocrine hormone levels. Studies were also eligible if they reported clinically relevant measures of estrogen deficiency related comorbidities, such as bone, vascular, metabolic or neurocognitive outcomes.

Exclusion criteria: (1) Full text unavailable; (2) Duplicate publication; (3) Non-original research (e.g., reviews or commentaries); (4) Interventions not derived from ginseng or studies unrelated to the ovarian context; (5) Incomplete reporting of key information.

Botanical nomenclature was verified against Plants of the World Online to ensure accuracy of the species name and origin for *P. ginseng* C.A. Mey. (https://powo.science.kew.org/; accessed 3 October 2025). Only studies describing clear extraction and preparation methods for ginseng extracts were included. For single compounds or high-purity constituents such as Rg1, Rb1, Rg3, and GPS, the source or purity assessment methods were required. Regarding multi-botanical formulas or composite preparations, we prioritized studies that performed mass spectrometric characterization and clearly identified major metabolites and representative active compounds. The final body of evidence included only studies of single-botanical drug *Panax ginseng*, its active compounds, and their metabolites; multi-botanical formulas were not considered.

## 3 Overview of ginseng and its plant-derived metabolites

Ginseng was first recorded in the *Shennong Bencao Jing* and categorized as a top-grade botanical drug, with a long history of clinical use (Chen, 2014). In *the Pharmacopoeia of the People’s Republic of China (Part I, 2025 edition),* ginseng is described as having multiple therapeutic functions, including tonifying vital qi, restoring the pulse and preventing collapse, tonifying the spleen and benefiting the lung, generating fluids and nourishing the blood, and calming the mind and enhancing cognition (National Pharmacopoeia Commission, 2020). This section focuses on modern pharmacological evidence regarding ginseng.

### 3.1 Plant-derived metabolites of ginseng

Current evidence indicates that the material basis of ginseng centers on triterpenoid saponins and polysaccharides (Ye et al., 2023; Zhang et al., 2023c), complemented by polyacetylenes and volatile small molecules that together form a multi-component, structurally diverse chemical profile (Liu et al., 2007; Cho, 2015).

Ginsenosides are the most representative active compounds of ginseng. Based on their steroid-like aglycone backbones, ginsenosides are broadly classified into protopanaxadiol (PPD)–type and protopanaxatriol (PPT)–type, differing systematically in both the number and positions of sugar moieties (Won et al., 2019). Representative PPD-type ginsenosides include Rb1, Rb2, Rc, Rd, Rg3, and CK, whereas PPT-type ginsenosides are exemplified by Rg1, Re, and Rh1 (Sharma and Lee, 2020). These ginsenosides vary in abundance, polarity, and bioavailability within ginseng, and these structural features directly influence their absorption and target engagement *in vivo*. Recent studies indicate that ginsenosides, including Rg1, Rg3, and CK, can delay ovarian functional decline, enhance follicular quality, and modulate sex hormone levels (He et al., 2021; Zhang et al., 2008; Ye et al., 2022), providing a mechanistic rationale for further evaluation of these active compounds in the context of ovarian aging.

GPS constitute another major class of active compounds, with more complex molecular architectures than ginsenosides (Zhang et al., 2023c). They are known for their immunomodulatory, antioxidant, and tissue-protective effects (Tao et al., 2023; Zhou R. et al., 2021). Their structures consist primarily of monosaccharides such as glucose, galactose and arabinose, and their pharmacological activity is closely linked to molecular weight and higher order conformation (Liu et al., 2021; Xu et al., 2024).

Additionally, ginseng contains polyacetylenes (e.g., panaxynol, panaxydol) (Knispel et al., 2013), as well as volatile small molecules, organic acids, amino acids, flavonoids, and other minor metabolites (Cho, 2015; Lee et al., 2022; Lee H. Y. et al., 2024; Yang et al., 2021). Although these compounds are typically present at low systemic concentrations in circulation and their direct pharmacological actions are still under active investigation, they may contribute to ginseng’s overall efficacy through synergistic modulation of cellular signaling pathways.

### 3.2 Pharmacokinetics and biotransformation

Ginseng and its active compounds exhibit marked component specificity and inter-individual variability across absorption, distribution, metabolism, and excretion (ADME) processes *in vivo* (Chen et al., 2018; Shen et al., 2013).

Ginsenosides, although pharmacologically active, are heavily glycosylated and relatively hydrophilic in their prototype forms, which results in generally low oral bioavailability (Leung and Wong, 2010; Wang et al., 2021). Upon oral administration, intestinal microbiota catalyze the stepwise deglycosylation of parent ginsenosides to produce metabolite type saponins such as CK (Kim, 2018). Compared with their prototypes, these metabolites exhibit enhanced membrane permeability, stronger plasma protein binding, and increased pharmacodynamic activity (Niu et al., 2013). This gut-driven transformation plays a pivotal role in systemic efficacy, resulting in a dual-track exposure pattern in which both prototypes and metabolites coexist in circulation. Notably, the extent and rate of this conversion vary substantially among individuals, influenced by factors such as gut microbiota composition, dietary patterns, and concomitant medications (Kim K. A. et al., 2015; Yang et al., 2011). This variability alters the ginsenoside metabolic spectrum and exposure, potentially contributing to heterogeneous therapeutic responses in interventions targeting ovarian aging. During distribution and elimination, ginsenosides and their metabolites are often handled by membrane transporters (e.g., P-glycoprotein) and may undergo enterohepatic circulation (EHC) (Hu et al., 2023). Pharmacokinetic parameters, such as maximum concentration (Cmax), area under the concentration–time curve (AUC), and elimination half-life (t_1_/_2_), differ substantially among individual compounds (Xie et al., 2019; Choi et al., 2020). These multi-component, multi-trajectory features should be considered when interpreting exposure–response relationships, dosing regimens, and therapeutic windows. In contrast, GPS, due to their high molecular weight and structural complexity, are less likely to be absorbed across the intestinal epithelium (Zhang J. et al., 2023). Therefore, their effects *in vivo* may be localized to the gut or mediated indirectly via immune regulation and related pathways (Kim et al., 2024).

Collectively, these features underscore the need, when evaluating ginseng for ovarian aging, to distinguish the bioavailability routes and levels of action of ginsenosides and polysaccharides.

### 3.3 Safety and interactions

Clinical and preclinical data suggest that ginseng and its active compounds are generally well tolerated within conventional dose ranges and treatment durations (Choi et al., 2020; Lee and Son, 2011; Fan et al., 2020), supporting their safety profile in chronic degenerative settings. The most commonly reported adverse events include mild insomnia, palpitations, and gastrointestinal discomfort, which are typically related to dose, reversible with time, and of low severity (Coon and Ernst, 2002; Li et al., 2023). Nonetheless, differences among preparation types, such as aqueous extracts, ethanolic extracts, total ginsenoside fractions, and multi-botanical formulations (Li et al., 2022; Kwang-Soon et al., 2008; Opuni et al., 2021), can substantially affect constituent profiles, dissolution behavior, and impurity burdens (Lee K.-Y. et al., 2024), which in turn may alter adverse event profiles and affect overall safety (Singh and Zhao, 2017).

Clinically, reported DDI with ginseng is most commonly observed with anticoagulants and antidiabetic agents. These interactions may occur via pharmacokinetic modulation (e.g., hepatic enzyme induction or inhibition) and pharmacodynamic synergy (e.g., antiplatelet or glucose-lowering effects) (Jiang et al., 2004; Gupta et al., 2017; Kim Y. S. et al., 2015). These risks warrant careful attention in polypharmacy settings. For long-term interventions targeting ovarian aging, particularly in patients with comorbidities or those receiving HRT, it is advisable to prespecify monitoring plans for plausible interaction-sensitive indices, such as coagulation parameters and blood glucose. Study protocols should also define stratified strategies for concomitant medications and clear discontinuation criteria to ensure safety and control.

Given the existing evidence gaps, special populations such as lactating women, women with fertility requirements, patients with major comorbidities, and those with hepatic or renal impairment should use ginseng at the lowest effective dose for the shortest necessary duration. Ongoing safety monitoring and careful assessment of risks and benefits based on available clinical data are recommended (Pitkälä et al., 2016).

## 4 Biological drivers of ovarian aging

Ovarian aging is driven by multiple interacting mechanisms and is characterized by a progressive decline in ovarian function and gradual depletion of the follicular reserve. Key contributors include oxidative stress, inflammation, apoptosis, dysregulated autophagy and disturbances in the HPO axis (Wang et al., 2023). These mechanisms not only occur independently but also influence one another, collectively shaping a gradual and largely irreversible aging trajectory. Understanding these mechanisms is essential for interpreting how candidate interventions may act on ovarian aging and for contextualizing the evidence base. To provide this context, we briefly outline these core drivers before synthesizing the ginseng-related evidence.

### 4.1 Oxidative stress and mitochondrial dysfunction

Oxidative stress is a key factor in aging and is characterized by an imbalance between reactive oxygen species (ROS) and antioxidants, which leads to cellular damage. The Oxidative Stress Theory of Aging (OSTA) suggests that accumulation of oxidative damage over time is a primary driver of aging processes (Edrey and Salmon, 2014). The ovary is particularly sensitive to oxidative stress because of its dynamic metabolic activity and the cyclic development and maturation of follicles, a combination that places substantial metabolic demands on ovarian tissue and increases susceptibility to oxidative injury. Oocytes are particularly vulnerable to ROS accumulation (Qin et al., 2025), which leads to mitochondrial dysfunction and a compromised cellular environment. Mitochondrial dysfunction, exemplified by decreased mitochondrial membrane potential (ΔΨm) and impaired autophagy regulation, alongside ROS buildup, plays a significant role in accelerating ovarian aging (Agarwal et al., 2005).

**FIGURE 1:**
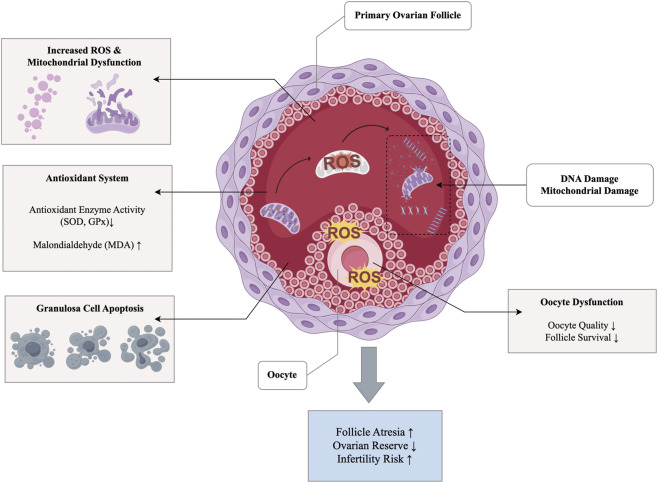
Mitochondrial dysfunction driven by oxidative stress in ovarian aging.

ROS are constantly generated as byproducts of cellular metabolism, and when their production exceeds the capacity of endogenous antioxidant defense systems, oxidative stress ensues, leading to cellular dysfunction and structural damage (Sies, 2015). In particular, the oocytes within primordial follicles are especially susceptible to ROS. Accumulation of ROS in primordial follicles can damage oocytes, triggering reproductive aging. Excessive ROS can directly damage oocyte DNA, mitochondria, and membranes, leading to apoptosis or functional inactivation, which accelerates follicular atrophy and depletion of the ovarian reserve (Song et al., 2024; Immediata et al., 2022; Lin and Wang, 2020). In aged mammalian models, ovarian ROS levels are significantly elevated, accompanied by delayed zona pellucida dissolution, disorganized cytoskeletal architecture, and increased granulosa cell apoptosis, hallmarks of oxidative injury (Hamatani et al., 2004; Tarín, 1996; Ben-Meir et al., 2015). Antioxidant enzymes such as superoxide dismutase (SOD) and glutathione peroxidase (GPx), along with ovarian antioxidant genes like Sod1, Sod2, Gpx1, and Gpx3, are downregulated in aging ovaries, which further weakens the defense mechanisms against oxidative stress (Lim and Luderer, 2011). When mitochondrial function is impaired, ROS production may enter a vicious cycle that perpetuates oxidative damage, disrupts energy metabolism and cellular homeostasis and further accelerates ovarian aging (Sena and Chandel, 2012; Picard and Turnbull, 2013). A classic model of ovarian oxidative stress involves the use of H_2_O_2_ to induce ovarian damage in mice. The resulting features closely resemble those observed in natural ovarian aging, indicating that oxidative stress not only accompanies aging but may serve as a key driver in the pathogenesis of ovarian dysfunction (Liang et al., 2024). These interrelated mechanisms are schematically summarized in Figure 1. These findings suggest that oxidative stress is not merely a byproduct of aging, but a key driving force in the process of ovarian aging.

### 4.2 Inflammaging and immune responses

**FIGURE 2:**
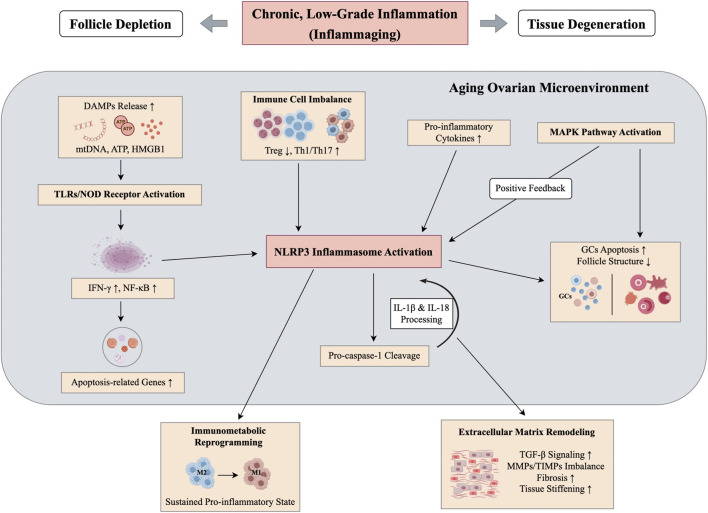
Inflammaging and immune dysregulation in the aging ovary.

Inflammation is an immune response to pathogens, tissue injury or harmful stimuli that normally serves to eliminate threats and promote repair (Medzhitov, 2008). However, in ovarian aging, this inflammation often becomes chronic, low-grade, and persistent, which disrupts tissue function and cellular integrity (Zhang et al., 2020; Lliberos et al., 2021). As aging progresses, the ovarian microenvironment becomes increasingly proinflammatory, with higher concentrations of cytokines such as tumor necrosis factor-α (TNF-α), interleukin-6 (IL-6) and interferon-γ (IFN-γ) (Bafor et al., 2024; Lliberos et al., 2020). This shift is accompanied by activation of a senescence-associated secretory phenotype, with sustained release of cytokines, chemokines and proteases, a pattern often referred to as inflammaging that contributes to follicular depletion and structural degeneration of the ovary (Zhang et al., 2020; Jiao et al., 2021) (Figure 2). Granulosa cells play a crucial role in maintaining the ovarian microenvironment. Chronic exposure to inflammatory mediators induces granulosa cell apoptosis, reduces proliferative capacity, and impairs follicle development (Crespo et al., 2010; Yamamoto et al., 2015; Alpizar and Spicer, 1994). Prolonged exposure to IFN-γ activates the NF-κB pathway in granulosa cells, upregulating pro-apoptotic genes and promoting cell injury (Zhao et al., 2022; Xiao et al., 2001). Inflammatory signals also recruit immune cells into ovarian tissue, disrupting immune homeostasis and amplifying local inflammatory responses, which leads to further damage (Al-Alem et al., 2015; Wu J. et al., 2015). As ovarian aging progresses, macrophage numbers increase and polarization shifts from anti-inflammatory (M2) to pro-inflammatory (M1) phenotypes, exacerbating the inflammatory microenvironment (Xiao et al., 2022). These changes activate multiple cytokine signaling pathways that alter oocyte and granulosa cell metabolism and survival, further accelerating ovarian aging (Dang et al., 2017; Peluso et al., 2001). Chronic inflammation not only impairs follicle quality but also promotes fibrotic remodeling, alters ovarian structure and disrupts normal follicle recruitment and ovulation (Lliberos et al., 2021; Ma et al., 2024).

From an immunological perspective, both innate and adaptive immune systems collaborate to maintain ovarian homeostasis. With aging, Treg cells decrease, while Th1 and Th17 cells become more prevalent. This imbalance promotes the production of proinflammatory cytokines and chemokines, further reducing immune tolerance and sustaining inflammation (Jiao et al., 2021; Deng et al., 2024). Endogenous damage-associated molecular patterns (DAMPs), including mitochondrial DNA, ATP, and HMGB1, can activate Toll-like receptors and NOD-like receptors in the absence of infection, triggering sustained NF-κB and MAPK signaling pathways and perpetuating low-grade inflammation (Liu Y. et al., 2020; Navarro-Pando et al., 2021; Liu et al., 2025). In ovarian aging, the NLRP3 inflammasome is activated, leading to the processing of pro-caspase-1 and the maturation of interleukin-1β (IL-1β) and interleukin-18 (IL-18), creating a positive feedback loop that accelerates granulosa cell apoptosis and follicular atresia (Lliberos et al., 2020; Navarro-Pando et al., 2021). Additionally, immunometabolic reprogramming stabilizes inflammaging, as M1 macrophages favor glycolysis and ROS production, while M2 macrophages rely on oxidative phosphorylation and fatty acid oxidation. These aging-related metabolic shifts help sustain pro-inflammatory states in immune cells, which further impair the follicular niche and promote ovarian aging (Zhang et al., 2020; Park et al., 2025). At the tissue level, inflammatory signaling via TGF-β and the imbalance between matrix metalloproteinases (MMPs) and their inhibitors contribute to extracellular matrix deposition and ovarian stromal stiffening, further impairing follicular recruitment and ovarian function (Isola et al., 2024). Additionally, upregulation of endothelial adhesion molecules and chemokines such as C-C motif chemokine ligand 2 (CCL2) and C-X-C motif chemokine ligand 10 (CXCL10) increases immune cell infiltration and prolongs inflammation, which further disrupts follicle recruitment and ovulation (Wang and Sun, 2022). In ovarian aging studies, key markers of inflammation, such as NLRP3-caspase-1-IL-1β axis activity, NF-κB phosphorylation, and the M1/M2 macrophage ratio, can serve as valuable indicators for evaluating the links between immune imbalance, inflammaging, and tissue remodeling. These evaluation methods provide critical insights into the mechanisms driving ovarian aging.

### 4.3 Apoptosis and autophagy dysregulation

**FIGURE 3:**
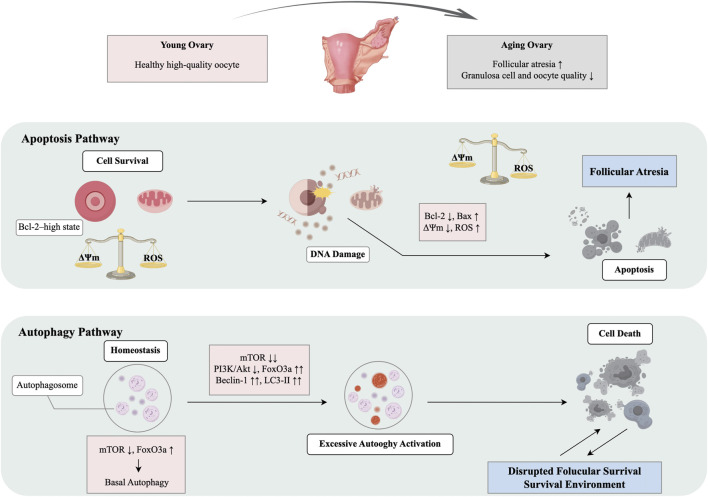
Crosstalk between apoptosis and autophagy in ovarian aging.

Apoptosis and autophagy are two key processes that contribute to follicular atresia, granulosa cell turnover, and oocyte quality control in the ovary (Zhou et al., 2019). During aging, the balance between these processes becomes disrupted, with increased apoptosis and dysregulated autophagy playing a significant role in ovarian aging (Jin et al., 2025; Peters et al., 2021; Tang et al., 2025). As ovaries age, granulosa cells and oocytes become more sensitive to both endogenous and exogenous stress signals. These cells exhibit reduced mitochondrial membrane potential (ΔΨm), increased ROS, and DNA damage, all of which trigger apoptotic pathways (Li C. et al., 2025; Martin et al., 2022; Smits et al., 2023). In aged ovaries, dysregulation of Bcl-2 family proteins, along with activation of p53 and caspase cascades, are hallmark features of heightened apoptosis (Kugu et al., 1998). In aged mouse ovaries, the pro-apoptotic protein Bax is upregulated, and the anti-apoptotic protein Bcl-2 is downregulated in granulosa cells, which accelerates follicular apoptosis and atresia (Yao et al., 2024). Autophagy, a protective mechanism that removes damaged organelles and misfolded proteins, plays a double-edged role in ovarian aging (Tang et al., 2025). While moderate autophagy helps maintain oocyte homeostasis (Nakashima et al., 2023), excessive autophagy, especially under oxidative stress or chemotherapy-induced injury, can promote cell death (Liang et al., 2024; Zhang et al., 2023d; Zhu et al., 2024). Key signaling nodes, such as PI3K–Akt–mTOR, regulate the balance between apoptosis and autophagy. In aged ovaries, reduced PI3K–Akt activity increases FoxO3a-mediated expression of autophagy genes, while mTOR inhibition sustains autophagy activity and disrupts the follicular niche (John et al., 2008; Li et al., 2024; Yu et al., 2011). In aged ovaries, changes in autophagy-related proteins like Beclin-1 and LC3-II highlight the imbalance between apoptosis and autophagy, making this dysregulation a crucial feature of ovarian aging (Navarro-Pando et al., 2021; Li et al., 2020). Thus, dysregulated apoptosis and autophagy are critical mechanisms driving ovarian aging and the depletion of the ovarian reserve (Figure 3).

### 4.4 Dysregulation of the HPO axis and imbalance of hormone-receptor expression

**FIGURE 4:**
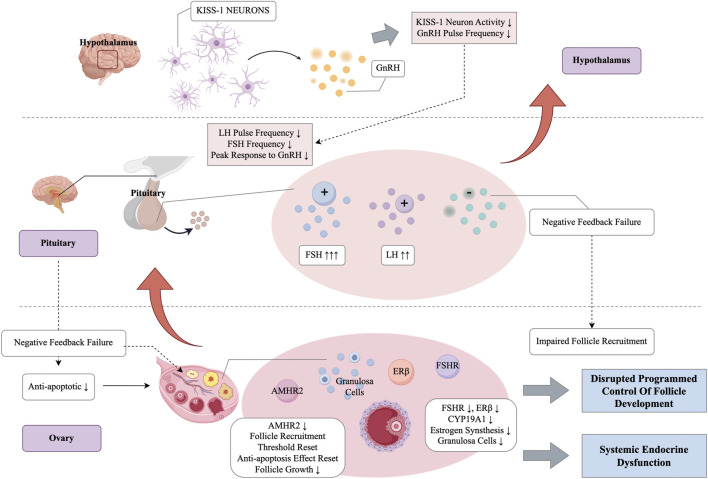
Dysregulation of the HPO axis and hormone–receptor expression in ovarian aging.

The maintenance of normal ovarian function depends on precise regulation within the HPO axis (Belchetz et al., 1978). However, with aging, feedback control in the HPO axis becomes disrupted. For instance, the frequency of gonadotropin-releasing hormone (GnRH) pulses declines, and the pituitary response to GnRH weakens. Additionally, the sensitivity of ovarian tissues to these hormones diminishes. These changes contribute to disordered follicle development and a decline in ovarian endocrine function (Hall et al., 2000; Jiang et al., 2015). In individuals with ovarian aging, serum levels of follicle-stimulating hormone (FSH) and luteinizing hormone (LH) increase, while estradiol (E_2_) decreases, indicating a loss of the normal negative feedback regulation (Nelson, 2009; Tepper et al., 2012). At the ovarian level, the expression of hormone receptors is also altered. Dysregulation of the FSH receptor (FSHR) and estrogen receptors (ERα and ERβ) impairs follicle recruitment and granulosa cell differentiation (Bramble et al., 2016; Xu X. L. et al., 2022; Couse et al., 2005). As circulating anti-Müllerian hormone (AMH) levels decline with age, or AMHR2 regulation becomes imbalanced, the recruitment threshold for early follicles is altered. This reduces their sensitivity to FSH, impairing ovarian function. Consequently, the maximal response of follicles to FSH diminishes, and the effective response window shortens, leading to reduced steroidogenesis and proliferative responses to FSH stimulation (Chang et al., 2013; van Rooij et al., 2005). In aged mouse ovaries, FSHR expression declines, which reduces granulosa cell sensitivity to FSH and affects aromatase (CYP19A1) expression and estradiol synthesis (Cuomo et al., 2018; Egbert et al., 2019; Devillers et al., 2019). Additionally, the receptor for LH and human chorionic gonadotropin (LHCGR) shows altered timing or premature upregulation in granulosa cells, leading to premature luteinization and impairing follicle selection and growth (Wu Y. G. et al., 2015). The reduced expression of ERβ in oocytes also diminishes estrogen’s anti-apoptotic effects and its support for follicle growth (Xu X. L. et al., 2022; Antonson et al., 2020; Haddad et al., 2020). These alterations in the HPO axis and receptor expression cause a loss of the finely tuned feedback mechanisms that are essential for normal follicle recruitment, selection, and ovulation, ultimately accelerating ovarian aging and depletion of the ovarian reserve (Figure 4).

## 5 Mechanism-guided evidence synthesis of ginseng interventions in ovarian aging

Ginseng, a traditional botanical drug, contains bioactive compounds such as ginsenosides, GPS, and polyacetylenes, which exert a variety of pharmacological effects. Studies *in vivo* and *in vitro* show that ginseng and its active compounds can protect ovarian structure and function through several mechanisms. These include modulating oxidative stress and mitochondrial function, regulating immune-inflammatory responses within the ovarian microenvironment, inhibiting apoptosis, promoting granulosa cell survival and autophagy balance, and modulating the HPO axis. These actions may delay the progression of ovarian aging or improve its phenotype, suggesting ginseng’s potential as an intervention.

### 5.1 Antioxidation and mitochondrial protection

Oxidative stress and mitochondrial dysfunction are central contributors to ovarian aging. Evidence suggests that ginseng and its active compounds may provide effective interventions to counter these processes, potentially ameliorating age-related damage in the ovary.

In a D-galactose (D-gal)-induced mouse model of ovarian dysfunction, Rg1 significantly reduced malondialdehyde (MDA) levels in ovarian tissue, reflecting a reduction in oxidative damage. It also enhanced the activity of key antioxidant enzymes, such as superoxide dismutase (SOD) and glutathione peroxidase (GPx), which are essential for maintaining redox balance. This restoration of oxidative defenses suggests that Rg1 can help the ovary recover from cellular damage. Histological analysis showed that Rg1 alleviated estrous cycle disruption and decreased the levels of senescence markers, including p53, p21, and p16. These results demonstrate that Rg1 not only mitigates oxidative damage but also combats cellular aging, improving ovarian function and fertility (He et al., 2017). *In vitro* studies further confirmed that Rg1 prevented stress-induced apoptosis in granulosa cells by preserving mitochondrial function and regulating mitophagy (Li X. et al., 2025). Similarly, Rb1 reduced ROS and MDA levels, restored mitochondrial membrane potential (ΔΨm), and activated the Akt-FoxO1 signaling pathway to improve mitochondrial dysfunction and counter oxidative damage (Zhou et al., 2022). This suggests that both Rg1 and Rb1 can work synergistically to improve mitochondrial health and reduce oxidative injury, critical factors in ovarian aging. Furthermore, in a nicotine-induced ovarian injury model, oral ginseng powder increased the number of healthy follicles, reduced atretic follicles, and increased the number of corpora lutea. The Ki-67 proliferation index was elevated in granulosa and theca cells, while the Bax/Bcl-2 ratio and cytochrome c (Cyt c) levels were reduced. These findings indicate that ginseng improves the ovarian microenvironment by alleviating oxidative stress and mitigating mitochondrial-dependent apoptosis (Faghani et al., 2022).

These results highlight the therapeutic potential of ginseng as an intervention to slow ovarian aging by enhancing antioxidant defenses and restoring mitochondrial function. However, much of the current evidence is inferred from oxidative stress indices and mitochondrial membrane potential (ΔΨm), although a subset of studies extends these readouts to ovary level phenotypes and endocrine indices, such as improved estrous cyclicity in D-gal models and increased healthy follicles and corpora lutea in nicotine-exposed mice. At the cellular level, granulosa cell stress assays further support preservation of mitochondrial function, including restoration of mitochondrial membrane potential (ΔΨm) together with measures of ATP production, but these short-term systems cannot on their own address oocyte competence or long-term functional reproductive outcomes. Direct links between mitochondrial energy metabolism, oocyte competence, and sustained reproductive outcomes remain limited. In addition, several commonly used models capture acute oxidative injury or toxicant induced ovarian dysfunction, which may not fully reflect the gradual and multifactorial course of physiological ovarian aging. Taken together, the strongest support for this mechanism comes from *in vivo* studies that connect oxidative stress and mitochondrial changes with ovary-level phenotypes, whereas evidence based mainly on short-term cell stress assays or isolated marker changes should be interpreted more cautiously. These findings support redox control and mitochondrial preservation as a plausible mechanistic node for ginseng-related interventions in ovarian aging.

### 5.2 Anti-inflammation and immunomodulation

Ginseng and its active compounds modulate inflammatory and immune processes within the ovarian microenvironment. Their actions converge on key features of the chronic low-grade inflammatory state and senescence-associated secretory phenotype (SASP) activation that characterize ovarian aging. The inflammatory phenotype in this context is not limited to elevated levels of a few cytokines but involves broader remodeling of resident immune cells, including macrophages, dendritic cells, natural killer cells and T cell subsets, together with a persistently amplified SASP signature (Zhang et al., 2020; Tang et al., 2023; Chen et al., 2022; Zhu et al., 2025).

Inflammasome activation drives ovarian inflammatory responses that contribute to age-related follicle depletion, and suppression of these processes may help delay ovarian aging and extend the reproductive window (Lliberos et al., 2020). In rat models of ovarian aging, rare ginsenosides known as heat transformed saponins (HTS) suppress p38 MAPK/NF-κB p65 signaling, lessening both inflammatory and oxidative stress burden. Combined *in vivo* and *in vitro* studies have identified a specific inhibitory effect of these saponins on the NLRP3 inflammasome, with reduced caspase-1 activation and lower maturation and secretion of interleukin 1β (IL-1β). This pattern is consistent with modulation of the NF-κB and NLRP3 axis as an important mechanism of inflammatory control within the ovarian microenvironment (Tao et al., 2024). In parallel, ginsenoside Rg3 specifically inhibits NLRP3 inflammasome activation, which reduces caspase-1 activation and decreases IL-1β maturation and secretion (Shi et al., 2020). Rare ginsenosides thereby dampen the NLRP3–caspase-1–IL-1β axis, which may in turn limit SASP amplification and inflammatory injury in ovarian tissue. Among ginseng metabolites, CK provides more direct insight into immunomodulation. In a lipopolysaccharide (LPS)-induced inflammatory model of RAW264.7 macrophages, CK selectively inhibits phosphorylation of AKT1 and downstream activation of IKKα, IKKβ and NF-κB signaling, leading to reduced expression of pro-inflammatory mediators such as IL-1β and TNF-α (Lee et al., 2019). In this setting CK reshapes innate immune responses by altering macrophage activation states and secretory profiles. Given the central role of ovarian macrophages in follicle recruitment, atresia and corpus luteum remodeling, the AKT1–IKK–NF-κB axis functions as a key signaling hub that links macrophage polarization, cytokine patterns and follicular fate, and it is a useful focus when evaluating ovarian targeted immunomodulatory actions of CK and related metabolites. In a three-dimensional ovarian follicle culture system, ginseng extract promoted follicle growth and functional maintenance, including hormone secretion, indicating a supportive effect on the ovarian microenvironment (Majdi Seghinsara et al., 2019).

Across both *in vivo* models and three-dimensional culture systems, ginseng combines antioxidative and anti-inflammatory effects with remodeling of the ovarian immune microenvironment. By modulating immune cell activity, cytokine networks and the SASP profile, ginseng and its active compounds may attenuate chronic inflammatory and immune activation in the ovary and help restore a microenvironment that is more favorable for long-term follicle survival. To avoid overinterpreting these findings, we distinguish between observations relevant to the ovary that suggest inflammatory remodeling of the ovarian microenvironment and mechanistic support at the pathway level derived from established immune cell systems. The latter is used to strengthen biological plausibility, but validation in the ovary is still needed. Accordingly, in the studies reviewed here, much of the mechanistic evidence for NF-κB and NLRP3 modulation is currently supported by macrophage-based inflammatory systems, including LPS-stimulated RAW264.7 cells and supporting luciferase reporter measurements, which strengthens pathway-level plausibility but still warrants deeper validation in ovary-relevant immune contexts. Evidence from ovarian aging models is emerging, for example, HTS reduced ovarian NF-κB p65 signaling and inflammatory mediators while improving estrous cyclicity and follicle related indices in cyclophosphamide induced ovarian failure models, yet the links between immune remodeling and functional reproductive outcomes remain insufficiently defined. In addition, three-dimensional follicle culture data support improved follicle growth and hormone secretion under ginseng extract exposure, but these systems lack local immune components and therefore cannot directly address immune cell dynamics. Collectively, evidence in this domain is anchored in ovary-relevant *in vivo* models and three-dimensional follicle culture systems, where modulation of inflammatory signaling aligns with improved ovarian indices and a more favorable follicle milieu. In contrast, pathway-level evidence derived mainly from macrophage-based inflammatory systems supports mechanistic plausibility, but it is less informative for ovary-specific immune remodeling and its links to long-term reproductive function. Therefore, future studies should prioritize ovary-based immune phenotyping and should directly characterize which immune cell populations are altered in the ovary, where they localize, and whether these changes differ across follicle stages and the corpus luteum. They should also test whether dampening inflammasome and SASP related signaling translates into maintained endocrine function and fertility outcomes during follow-up in appropriately controlled *in vivo* designs.

### 5.3 Anti-apoptosis and autophagy balance

During ovarian aging, enhanced apoptosis of granulosa cells together with dysregulated autophagy drive follicular atresia and a progressive decline in ovarian reserve. Active compounds from ginseng, represented by Rg1, Rg3, Rb1, CK and GPS, mainly act through coordinated regulation of the mitochondrial apoptotic pathway and autophagy signaling. In this way they reduce the apoptotic burden on granulosa cells and help preserve their synthetic and proliferative functions.

Preclinical studies have identified convergent regulatory effects of ginsenosides such as Rg1, Rb1 and Rg3 on mitochondrial apoptosis and autophagy balance. In a hydrogen peroxide induced oxidative stress model of granulosa cells, Rg1 preserved mitochondrial function and suppressed excessively activated, deleterious mitophagy, which lowered the apoptosis rate of granulosa cells. At the molecular level, Rg1 treatment was associated with downregulation of Bax, upregulation of Bcl-2 and reduced cleaved Caspase-3, together with concordant changes in LC3 and p62 expression, a pattern consistent with appropriately regulated autophagic flux (Li X. et al., 2025). In a D-gal-induced mouse model of ovarian aging, Rg1 further downregulated Akt, mTOR and S6K at both protein and mRNA levels and increased expression of the autophagy related marker LC3-II. This profile is in line with involvement of the PI3K–Akt–mTOR autophagy pathway in the regulation of ovarian function and coincides with improvement in estrous cycle disturbances and attenuation of histological ovarian damage (Liu et al., 2020a). A separate study extended these observations to systemic aging phenotypes. Rg1 reduced the expression of senescence-associated proteins p53, p21 and p16 in ovarian tissue, with concurrent improvement in estrous cyclicity, a more physiological sex hormone profile and less histological damage, indicating that Rg1 can also buffer apoptotic stress on granulosa cells through combined antioxidative and anti-inflammatory actions (He et al., 2017). Data on Rg3 and Rb1 from different experimental models further emphasize shared features of the mitochondrial apoptosis axis. In a human granulosa cell model, Rg3 markedly inhibited mitochondria-dependent apoptosis, reduced the proportion of apoptotic cells and increased the Bcl-2 to Bax ratio, while limiting cytochrome c release. These changes were accompanied by lower ROS levels, linking its anti-apoptotic action to relief of upstream oxidative stress (Zhou P. et al., 2021). In a cyclophosphamide induced model of ovarian aging, Rb1 corrected the imbalance in Bcl-2 and Bax expression, reduced granulosa cell apoptosis and exerted protective effects on ovarian structure and function (Tang et al., 2014). CK and GPS provide complementary evidence for granulosa cell protection from the perspectives of inflammation driven apoptosis and preservation of proliferative capacity. Consistent with the pathway level evidence summarized above, in LPS-stimulated RAW264.7 macrophages, CK suppresses AKT1 phosphorylation and downstream activation of IKKα and IKKβ and NF-κB signaling, supporting the concept that attenuating inflammatory drive may indirectly reduce granulosa cell apoptosis within an aging-like ovarian microenvironment (Lee et al., 2019). GPS primarily confers functional protection by maintaining the proliferative capacity and synthetic activity of granulosa cells. Studies have reported that GPS enhances DNA synthesis in granulosa cells and counteracts stress induced proliferation arrest (Feng et al., 2007), and promotes cell proliferation with upregulation of proliferation related genes such as PCNA, CDC42 and CDK2 (Chen et al., 2025). These findings indicate that GPS helps sustain granulosa cell renewal and functional integrity during follicular development and, beyond limiting apoptosis alone, provides cellular replenishment and functional support that contribute to the maintenance of ovarian reserve.

Within this mechanistic domain, ginsenosides represented by Rg1, Rg3 and Rb1 mainly act through refined regulation of the mitochondrial apoptotic pathway and autophagy signaling to directly reduce granulosa cell death. CK mitigates inflammatory signaling and thereby offers immunological support for limiting inflammation driven apoptosis. GPS enhances synthetic and proliferative capacity and in doing so stabilizes the granulosa cell pool and supports preservation of ovarian reserve. In summary, support for this domain is derived largely from ovary-relevant *in vivo* models, where reduced granulosa cell apoptosis and autophagy marker patterns consistent with a balanced autophagy state are accompanied by improved follicle and endocrine indices. In contrast, evidence based mainly on short-term granulosa cell stress paradigms and static marker measurements remains more preliminary for judging whether these pathways can preserve follicle survival and fertility-relevant outcomes over time. However, the current evidence base is dominated by short-term injury models and marker-centered outcome measures, including apoptosis markers and autophagy-associated protein levels assessed in granulosa cell stress paradigms or chemotherapy-related ovarian injury settings. Such designs support mechanistic plausibility but still leave two unresolved translational questions: first, whether these pathways are engaged in a follicle stage dependent manner during physiological aging, and second, whether their modulation is sufficient to preserve follicle survival, endocrine function, and ultimately fertility relevant outcomes. Moreover, reliance on static marker abundance rather than evidence that reflects autophagic flux, together with limited dose response characterization and scarce exposure information, constrains interpretation of how mitochondrial apoptosis and autophagy regulation map onto ovarian concentrations attainable *in vivo*. To strengthen causal interpretation, future work should test pathway necessity in ovary-relevant settings by directly assessing granulosa cells, oocytes and ovarian stromal cells, determining whether effects differ across follicle stages and the corpus luteum, and linking apoptosis and autophagy modulation to functional outcomes such as follicle viability, hormone levels and fertility related endpoints, while incorporating exposure guided dosing wherever feasible.

### 5.4 Modulation of the HPO axis and hormone levels

Ginseng and its active compounds, particularly Rg1 and GPS, have demonstrated the ability to enhance receptor expression and promote follicle development in ovarian aging models. These effects are accompanied by improvements in hormone profiles and antioxidative status, which help recalibrate the HPO axis.

In a D-gal-induced mouse model of ovarian aging, Rg1 treatment resulted in more follicles and no signs of vacuolation, while the D-gal group exhibited fewer follicles and significant ovarian damage. E_2_ levels increased, whereas FSH levels decreased. Furthermore, antioxidant enzymes such as superoxide dismutase (SOD) and catalase (CAT) were upregulated. These results suggest that Rg1 improves sex hormone levels and alleviates oxidative stress, thereby contributing to the restoration of ovarian function and overall health (Liu et al., 2020b). In another ovarian aging model, Rg1 significantly upregulated FSH receptor (FSHR) expression in granulosa cells, accompanied by increased serum E_2_ and anti-Müllerian hormone (AMH) levels, while FSH levels decreased. Follicular development improved, with a higher number of follicles in the Rg1 group compared to controls, and fertility outcomes were enhanced. This suggests that Rg1 boosts ovarian responsiveness to gonadotropins, thereby restoring a more youthful hormonal profile and enhancing reproductive function (He et al., 2017). *In vitro*, ginseng extract applied to mouse preantral follicles increased the expression of FSHR and PCNA mRNA. This was associated with improved follicle survival, antrum formation, and metaphase II (MII) maturation rates. Additionally, culture supernatants from treated follicles showed elevated E_2_ and progesterone (P4) levels, while oocyte ROS levels were reduced. These results support the idea that ginseng compounds directly modulate ovarian receptor expression and secretory function, promoting healthier ovarian function (Majdi Seghinsara et al., 2019). GPS further enhances ovarian function by regulating steroidogenesis and second-messenger signaling. In bovine granulosa cells, GPS enhanced E_2_ secretion and increased the levels of steroidogenic enzymes, such as CYP11A1, CYP19A1, 3β-HSD, and STAR. This suggests that GPS promotes estrogen biosynthesis, contributing to the restoration of hormone balance in the ovarian microenvironment (Chen et al., 2025). In a rat *in vitro* human chorionic gonadotropin (hCG) stimulation system, GPS suppressed progesterone production in luteal cells and enhanced progesterone secretion in granulosa cells. It also increased cyclic AMP (cAMP) levels, suggesting bidirectional modulation of ovarian responsiveness downstream of the HPO axis (Sun et al., 2007). In a rat model of estrogen decline induced by ovariectomy, intragastric administration of ginseng decoction prolonged the estrous phase, increased the uterine index, and raised serum E_2_ levels while lowering LH. Metabolomic analysis revealed significant changes in several biomarkers, with pathway analysis indicating alterations in steroid hormone metabolism, fatty acid biosynthesis, the tricarboxylic acid cycle, and tryptophan metabolism. These findings suggest that ginseng corrects system-level metabolic changes associated with estrogen loss, offering a promising approach to mitigating the effects of ovarian aging (Lin et al., 2018).

Collectively, these findings indicate that ginseng and its active compounds function as multi-level modulators of the HPO axis, integrating improved receptor expression, steroidogenesis, antioxidant defenses, and systemic metabolism to partially rejuvenate the ovarian microenvironment and mitigate the endocrine and reproductive consequences of ovarian aging. However, the current support relies largely on changes in circulating hormones together with ovarian receptor and steroidogenic markers, while evidence directly characterizing hypothalamic or pituitary signaling responses remains scarce. On balance, the strongest support for HPO-axis modulation comes from *in vivo* studies that combine circulating hormone shifts with ovary-level receptor and steroidogenic changes, and, where reported, improvements in cycle regularity or ovulation-related measures. In contrast, evidence is less definitive when it is limited to serum hormone changes and ovarian marker expression without direct characterization of hypothalamic or pituitary signaling dynamics. *In vitro* follicle and granulosa cell data help substantiate direct ovarian actions, but differences in preparation definition and exposure reporting constrain cross-study interpretation. Accordingly, future studies should link endocrine shifts to functional endpoints such as ovulation and fertility outcomes, and integrate ovary-relevant receptor and steroidogenic assessments with exposure-informed study designs to clarify dose–response relationships along the HPO axis.

### 5.5 Therapeutic implications of ginseng in long-term complications associated with ovarian aging

Chronic estrogen deficiency due to ovarian aging is a major contributor to various long-term comorbidities affecting multiple organ systems. Clinical management of ovarian aging should not be limited to immediate fertility or menstrual concerns, but should also incorporate long-term strategies to prevent downstream comorbidities. Accordingly, structured follow-up should be considered for key clinical measures closely linked to these risks, such as bone health indicators relevant to osteoporosis prevention, glycemic control measures and parameters related to metabolic syndrome and cardiovascular risk markers, to integrate symptom management with comorbidity prevention. Given the central role of estrogen decline in skeletal remodeling, accelerated bone loss and osteoporosis risk often represent an early, clinically relevant, and preventable consequence of ovarian aging. In the skeletal system, imbalances in RANKL/OPG, combined with oxidative stress and mitochondrial dysfunction, contribute to bone loss and increase the risk of fragility fractures (Zhao et al., 2025; Azizieh et al., 2019; Zhang C. et al., 2023). In the cardiovascular system, reduced endothelial nitric oxide (NO) bioavailability, adverse lipid profiles, and persistent low-grade inflammation accelerate atherosclerosis development (Nair et al., 2021; Somani et al., 2019). In the nervous system, diminished estrogenic support for synaptic plasticity and mitochondrial energy metabolism, compounded by oxidative and inflammatory stress, heightens the risk of cognitive decline and related impairments (Hara et al., 2015; Lejri et al., 2018; McCarthy and Raval, 2020). At the metabolic and muscular levels, estrogen deficiency associated with ovarian aging predisposes to insulin resistance and fat redistribution and disrupts mitochondrial homeostasis. These alterations are closely linked to the development of metabolic syndrome, non-alcoholic fatty liver disease (NAFLD) and phenotypes such as sarcopenia and frailty (Eng et al., 2023; Camon et al., 2024; Feng et al., 2024; Chen et al., 2023).

In the skeletal system, the ginseng metabolite CK inhibited RANKL-induced osteoclastogenesis and reduced oxidative stress in ovariectomized (OVX) models, leading to improved bone mass and trabecular microarchitecture (Ding et al., 2023). Similarly, Rc increased bone mass and improved bone structure in OVX mice (Yang et al., 2022), while Re suppressed osteoclast differentiation, further indicating that ginseng compounds can effectively limit bone loss (Park et al., 2016). In the cardiovascular system, Rb1 enhanced nitric oxide (NO) signaling, thereby protecting endothelial function via the GPER–PI3K–Akt pathway (Yang et al., 2019). Rg1 further promoted endothelial nitric oxide synthase (eNOS) activity, which increased NO production and improved vascular reactivity, reinforcing its potential as a therapeutic target for postmenopausal atherogenesis (Zhang et al., 2025). In the nervous system, Rg1 improved learning and memory, reduced hippocampal neuronal apoptosis, and lowered the accumulation of pathological proteins in models of vascular cognitive impairment and Alzheimer’s disease. These neuroprotective effects were associated with the activation of estrogen-related signaling pathways, including GPR30 (Shen et al., 2021; Li et al., 2016). Moreover, long-term administration of ginseng water extract reshaped the gut microbiota in naturally aged mice, and fecal microbiota transplantation from ginseng-treated donors alleviated markers of age-related brain damage. This highlights the potential neuroprotective effects of ginseng, which may be mediated through modulation of the microbiota–gut–brain axis (Lin et al., 2024). In terms of metabolism and muscle function, Rb1 activated AMP-activated protein kinase (AMPK), improving insulin sensitivity and correcting lipid metabolism (Shen et al., 2015; Zou et al., 2022). CK reduced inflammation, alleviated hepatic steatosis, and improved insulin resistance in models of high-fat diet-induced obesity, type 2 diabetes (T2DM), and NAFLD (Xu J. et al., 2022; Wei et al., 2015). Additionally, Rc mitigated muscle atrophy induced by oxidative stress and promoted mitochondrial biogenesis, suggesting that ginseng compounds provide protection across a broad spectrum of aging-related disorders, from sarcopenia to frailty (Kim et al., 2023).

Collectively, these studies suggest that ginseng-related compounds may mitigate a range of estrogen deficiency–associated systemic phenotypes, spanning bone remodeling, endothelial function, neurocognitive performance, and metabolic homeostasis. However, much of the evidence is derived from OVX models or organ-specific disease models, and the reported benefits are often based on heterogeneous outcome measures rather than clinically anchored endpoints. In practical terms, the strongest support comes from *in vivo* models where ginseng-related interventions improve organ-level functional measures, such as bone structural parameters, vascular function, metabolic indices, or cognition-related performance. In contrast, when findings are based on diverse measures across different OVX or organ-specific disease models, interpretation requires more context, and cross-model generalization to systemic benefit in ovarian aging is naturally more constrained. Accordingly, future work should prioritize designs that incorporate clinically meaningful outcomes such as bone density and strength, vascular function and atherosclerotic burden, cognition-related performance, and metabolic indices, while also clarifying dose–exposure relationships and safety considerations under real-world polypharmacy conditions.

## 6 Limitations and future research prospects

Although the evidence base remains predominantly preclinical, it is still useful to consider translational feasibility alongside study limitations. Reported doses, routes, and treatment durations vary widely across animal and cell systems, which makes direct extrapolation to clinical use inappropriate; accordingly, translational interpretation should place greater weight on patterns that are reproducible across models and aligned with ovary-level phenotypes. For orally administered ginsenosides, bioavailability is often limited and variable, and differences in processing and preparation can further diversify exposure profiles, so studies with traceable and chemically characterized preparations provide a more reliable basis for comparison. Safety also becomes central when long-term use is envisioned, particularly in women with comorbidities and concomitant medications, and efficacy claims should be interpreted together with reported adverse events and plausible DDI considerations. These points provide a practical context for the limitations summarized below. Current evidence on ginseng’s effects in ovarian aging is constrained by several factors. In particular, incomplete reporting in primary studies can further affect interpretation. As summarized in Supplementary Table S2, key details were sometimes unclear or insufficiently detailed, most often involving vehicle or solvent control reporting in cell-based experiments and the definition and characterization of the tested preparations. In the *in vivo* literature, random allocation was reported, whereas blinding was rarely reported, which limits confidence in effect size estimates for bias sensitive outcomes and may contribute to variability across studies. Variations in the botanical source and preparation methods can lead to inconsistent concentrations of active compounds, which complicates comparisons across studies. Additionally, the variability in dosing regimens, treatment durations, and administration routes complicates the synthesis of findings. For example, the conversion of parent ginsenosides into metabolites during oral administration is influenced by factors such as gut microbiota, diet, and membrane transporters, which differ across populations, affecting bioavailability and pharmacological outcomes (Kim K. A. et al., 2015; Kim, 2013). Across the field, many studies also emphasize marker-based outcomes, whereas functional reproductive endpoints and clinically meaningful systemic outcomes are less consistently reported.

To address these limitations, future research should focus on improving the standardization of ginseng preparations, including the establishment of traceable sourcing, processing methods and chemical fingerprints for active compounds. It is essential to standardize the reporting of composition, dosing, and administration schedules to improve cross-study comparability. When feasible, studies should report *in vivo* exposure information, particularly for oral interventions, to clarify how the administered dose relates to circulating parent ginsenosides and key metabolites. Additionally, the relationship between different administration routes and *in vivo* exposure should be further clarified. Oral studies, in particular, should measure both parent ginsenosides and key metabolites in plasma and ovarian tissue, while accounting for factors like microbiome composition and diet. Finally, large-scale multicenter randomized controlled trials should be conducted in defined populations, such as women with DOR or POI, and should incorporate extended follow-up to capture long-term reproductive and systemic outcomes, including adherence and safety data, thereby providing a more robust evidence base for clinical guidelines. Where long-term use is envisioned, study designs should incorporate prespecified safety surveillance and a structured plan to assess potential DDI.

## 7 Conclusion

Ovarian aging is a complex and multifactorial process, involving the interplay of multiple biological mechanisms. Ginseng, particularly its bioactive compounds like saponins and polysaccharides, addresses these issues by reducing oxidative stress, enhancing mitochondrial function, and regulating energy metabolism, while also suppressing chronic inflammation and modulating immune responses. However, the current evidence is predominantly preclinical and heterogeneous, and many findings are based on surrogate molecular and endocrine measures rather than clinically meaningful reproductive outcomes. At the ovarian level, ginseng compounds promote receptor expression and steroidogenic enzymes, supporting the restoration of hormonal balance. These compounds also protect granulosa cells by preventing apoptosis, regulating autophagic processes and maintaining the integrity of the follicular niche. Beyond ovarian effects, ginseng has been reported in preclinical studies to improves endothelial function, restores bone metabolism, and modulates the gut microbiota, which may reduce long-term risks like osteoporosis, atherosclerosis, and cognitive decline, but the translational relevance of these multisystem benefits still warrants cautious interpretation and further clinical evaluation. These findings warrant further investigation into the molecular underpinnings of ginseng’s action and its potential for clinical translation in managing ovarian aging and its associated comorbidities. Future research should focus on optimizing dosing regimens with exposure-guided strategies informed by pharmacokinetics and microbiota mediated biotransformation, clarifying the mechanisms of action in ovary-relevant models with stage-stratified designs, and establishing robust approaches to long-term efficacy and safety evaluation, with continued monitoring of adverse events and assessment of outcomes and clinical measures that are prone to drug interaction effects in women with age-related or pathological ovarian decline, particularly among those receiving multiple concomitant medications.
