# Supplementary material for: Ginseng and its active compounds in ovarian aging: mechanistic basis and translational prospects
Source: Front Pharmacol. 2026 Jan 26;17:1755093. doi: 10.3389/fphar.2026.1755093 (PMC12884172; doi:10.3389/fphar.2026.1755093)
Supplement: Supplementary file 1 [file Table1.docx]

Supplementary Material

**Supplementary Table1 Literature search method .**

| Pubmed | #1 ("Ovarian Aging"[MeSH Terms] OR "Ovarian Insufficiency, Premature"[MeSH Terms] OR ovarian aging[Title/Abstract] OR ovarian senescence[Title/Abstract] OR diminished ovarian reserve[Title/Abstract] OR DOR[Title/Abstract] OR premature ovarian insufficiency[Title/Abstract] OR POI[Title/Abstract] OR primary ovarian insufficiency[Title/Abstract] OR premature ovarian failure[Title/Abstract] OR POF[Title/Abstract])  #2 (Ginseng[MeSH Terms] OR Panax[MeSH Terms] OR ginsenosides[MeSH Terms] OR Panax ginseng[Title/Abstract] OR ginseng[Title/Abstract] OR ginsenoside*[Title/Abstract] OR Rg1[Title/Abstract] OR Rb1[Title/Abstract] OR Rg3[Title/Abstract] OR ginsenoside Rg1[Title/Abstract] OR ginsenoside Rb1[Title/Abstract] OR ginsenoside Rg3[Title/Abstract] OR compound k[Title/Abstract] OR ginseng polysaccharide*[Title/Abstract])  #1 AND #2  Search date: 15 October 2025 |
| --- | --- |
| Embase | #1 'ovarian aging'/exp OR 'ovarian senescence':ti,ab OR 'diminished ovarian reserve':ti,ab OR DOR:ti,ab OR 'premature ovarian insufficiency'/exp OR POI:ti,ab OR 'primary ovarian insufficiency':ti,ab OR 'premature ovarian failure':ti,ab OR POF:ti,ab  #2 'ginseng'/exp OR 'panax'/exp OR 'ginsenosides'/exp OR 'panax ginseng':ti,ab OR ginseng:ti,ab OR ginsenoside*:ti,ab OR Rg1:ti,ab OR Rb1:ti,ab OR Rg3:ti,ab OR 'ginsenoside Rg1':ti,ab OR 'ginsenoside Rb1':ti,ab OR 'ginsenoside Rg3':ti,ab OR 'compound k':ti,ab OR 'ginseng polysaccharide*':ti,ab  #1 AND #2  Search date: 15 October 2025 |
| CNKI | SU=( 'ovarian aging' OR 'diminished ovarian reserve' OR DOR OR 'premature ovarian insufficiency' OR 'primary ovarian insufficiency' OR POI OR 'premature ovarian failure' OR POF )  AND  SU=( 'Panax ginseng' OR ginseng OR ginsenosides OR ginsenoside* OR Rg1 OR Rb1 OR Rg3 OR 'ginsenoside Rg1' OR 'ginsenoside Rb1' OR 'ginsenoside Rg3' OR 'compound K' OR 'ginseng polysaccharide*' )  Search date: 15 October 2025 |

**Note:** (1) *The Pharmacopoeia of the People’s Republic of China* (Part I, 2025 edition) was consulted for official botanical identification and quality-control information on *Panax ginseng* C.A. Mey. Reference lists of relevant reviews and included studies were also screened to identify additional eligible records. (2) Mechanism-oriented terms (e.g., oxidative stress, inflammaging) were used in supplementary searches to facilitate retrieval of pathway-focused studies.
