# Supplementary material for: Ginseng and its active compounds in ovarian aging: mechanistic basis and translational prospects
Source: Front Pharmacol. 2026 Jan 26;17:1755093. doi: 10.3389/fphar.2026.1755093 (PMC12884172; doi:10.3389/fphar.2026.1755093)
Supplement: Supplementary file 2 [file Table2.docx]

Supplementary Material

**Supplementary Table 2 Simplified reporting-based checklist of key items sensitive to bias.**

| **Study** | **Evidence type** | **Control groups reported** | **Vehicle and vehicle control reported** | **Dose or final concentration reported** | **Route, frequency, duration, and timing reported** | **Intervention identifiers reported** | **Overall notes**  **(optional)** |
| --- | --- | --- | --- | --- | --- | --- | --- |
| He et al. (2017). | *In vivo* (C57BL/6 female mice). | R | R | R | R | R | - |
| Li et al. (2025). | *In vitro* (KGN human ovarian granulosa cells). | R | P | R | R | R | Vehicle control is clearly specified (0.1% DMSO), but Rg1 solvent or stock preparation is not explicitly described. |
| Zhou et al. (2022). | *In vivo* (ICR female mice; young and old) .  *In vitro* (KGN human granulosa cell line and primary human granulosa-lutein cells, hGL). | R | P | R | R | R | Vehicle reporting is clear *in vivo*, whereas *in vitro* vehicle matching for the DMSO-based stock is not explicitly described. |
| Faghani et al. (2022). | *In vivo* (adult female NMRI mice, 8–10 weeks old). | R | P | R | R | R | PG is described as “PG powder” with a stated source and solvent, but no chemical standardization details. |
| Tao et al. (2024). | *In vivo* (Sprague–Dawley rats, female, 6-week-old) .  *In vitro* (KGN human granulosa-like cells). | R | P | R | R | R | Oral vehicle matching was not explicitly described for each group; oral distilled water was specified only for the control group. |
| Shi et al. (2020). | *In vitro* (THP-1 human monocyte cells; HEK293T human embryonic kidney cells; BMDMs mouse bone marrow-derived macrophages; J774A.1 mouse macrophage cells; RAW264.7 and RAW264.7-ASC mouse macrophage cells).  *In vivo* (C57BL/6J mice). | R | R | R | R | R | Not an ovarian model. This study provides mechanism-supporting **indirect evidence** that ginsenoside Rg3 downregulates NLRP3 inflammasome activation in macrophage-driven innate immune responses and in mouse *in vivo* models, supporting an inflammaging-oriented interpretation. |
| Lee et al. (2019). | *In vitro* (RAW264.7 mouse macrophage cells; HEK293 human embryonic kidney cells). | R | P | R | R | R | Vehicle information for Compound K was partially reported: source and purity were stated, but the solvent and vehicle-only control were not specified.  Not an ovarian model. This study offers mechanism-supporting **indirect evidence** that Compound K can suppress inflammatory signaling in mouse macrophage cells and human signaling reporter systems, which is relevant for interpreting inflammation-related pathways. |
| Majdi et al. (2019). | *In vitro* (preantral follicles isolated from 14-day-old female NMRI mice; 3D alginate culture). | R | P | R | R | R | *In vitro* vehicle matching is not explicitly described: PGE stock was prepared in DMSO, but a DMSO-matched vehicle control is not clearly reported. |
| Liu et al. (2020a). | *In vivo* (BALB/c female mice, 6–8 weeks). | R | R | R | R | R | - |
| Zhou et al. (2021). | *In vitro* (KGN human ovarian granulosa cell line). | R | NR | R | R | R | Vehicle for Rg3 preparation and an explicit matched vehicle control condition are not reported. |
| Tang et al. (2014). | *In vivo* (Wistar female rats). | R | P | R | R | NR | Saline gavage is stated for the model group, but the vehicle used for Rb1 gavage is not explicitly reported, and the control group is described as receiving no intervention with no matched gavage control.  Rb1 identifiers are not reported. Supplier, purity, and batch or catalog information are not provided in the reagent information available. |
| Feng et al. (2007). | *In vitro* (Wistar rat oocytes; Wistar rat ovarian granulosa cells). | R | NR | R | R | NR | The solvent or vehicle used to prepare ginseng polysaccharides was not explicitly reported, and a strictly matched vehicle control condition was not described.  GPS source, purity, and preparation details were not reported. |
| Chen et al. (2025). | *In vitro* (primary bovine ovarian granulosa cells). | R | R | R | R | R | - |
| Liu et al. (2020b). | *In vivo* (BALB/c female mice). | R | R | R | R | R | - |
| Sun et al. (2007). | *In vitro* (adult female rat-derived ovarian luteal cells, granulosa cells, and oocytes). | R | P | R | R | NR | The control group was explicitly described as receiving PBS. In the other *in vitro* experiments, the solvent or vehicle used to prepare the ginseng polysaccharide was not specified. The ginseng polysaccharide was described only as a laboratory gift with a stated stock concentration, and supplier information, catalog number, batch number, purity, and compositional characterization were not provided. |
| Lin et al. (2018). | *In vivo* (Sprague-Dawley female rats). | R | P | R | P | R | The gavage vehicle is described for the distilled water groups and the ginseng decoction, but the vehicle and administration details for estradiol valerate are not specified.  Route and duration are reported as intragastric administration for 8 weeks, but dosing frequency is not explicitly stated. |

**Note:** This table summarizes reporting completeness for key bias sensitive items and is not intended as a subjective risk rating. Codes are defined as follows: **R**, reported with sufficient detail to allow verification or reproducibility; **P**, partially reported, with one or more key details missing; **NR**, not reported or not determinable from the article text and supplementary materials. In the vehicle domain, “vehicle” includes the solvent where applicable. “Intervention identifiers” refer to the intervention name and form, and source or supplier information when available.
